# Supplementary material for: Self-assembly of hybrid 3D cultures by integrating living and synthetic cells
Source: Nat Commun. 2025 Dec 10;16:11073. doi: 10.1038/s41467-025-66789-3 (PMC12698700; doi:10.1038/s41467-025-66789-3)
Supplement: Supplementary file 1 — Supplementary Information File [file 41467_2025_66789_MOESM1_ESM.pdf]

Supplementary Information

Title: Self-assembly of hybrid 3D cultures by integrating living and synthetic cells

Authors: Nils Piernitzki[1,2,3], Ning Gao[4], Gilles Gasparoni[5], Louisa M. Krauß[6], Julia Schulze-Hentrich[5], Michael Dustin[7], Bianca Schrul[3,6,8], Balázs Győrffy[9,10,11], Stephen Mann[4,12], Oskar Staufer[1,2,3,4,8*]

*Corresponding author: Oskar Staufer, oskar.staufer@leibniz-inm.de

Affiliations:

[1]INM - Leibniz Institute for New Materials, Campus D2 2, 66123 Saarbrücken, Germany
[2]Helmholtz Institute for Pharmaceutical Research Saarland, Helmholtz Center for Infection Research, Campus E8 1, 66123 Saarbrücken, Germany
[3]Center for Biophysics, Saarland University, Campus Saarland, 66123 Saarbrücken, Germany
[4]Max Planck Bristol Centre for Minimal Biology, Cantock's Close, Bristol BS8 1TS, United Kingdom
[5]Department of Genetics/Epigenetics, Faculty NT, Saarland University, Saarbrücken, Germany
[6]Medical Biochemistry and Molecular Biology, Center for Molecular Signaling (PZMS), Faculty of Medicine, Saarland University, 66421, Homburg/Saar, Germany
[7]Kennedy Institute of Rheumatology, University of Oxford, Oxford, UK
[8]PharmaScienceHub (PSH), Saarbrücken, Germany
[9]Department of Bioinformatics, Semmelweis University, 1094 Budapest, Hungary
[10]Dept. of Biophysics, Medical School, University of Pecs, H-7624, Pecs, Hungary
[11]Cancer Biomarker Research Group, Institute of Molecular Life Sciences, HUN-REN Research Centre for Natural Sciences, H-1117, Budapest, Hungary
[12]Centre for Protolife Research and Centre for Organized Matter Chemistry, School of Chemistry, University of Bristol, Bristol BS8 1TS, UK

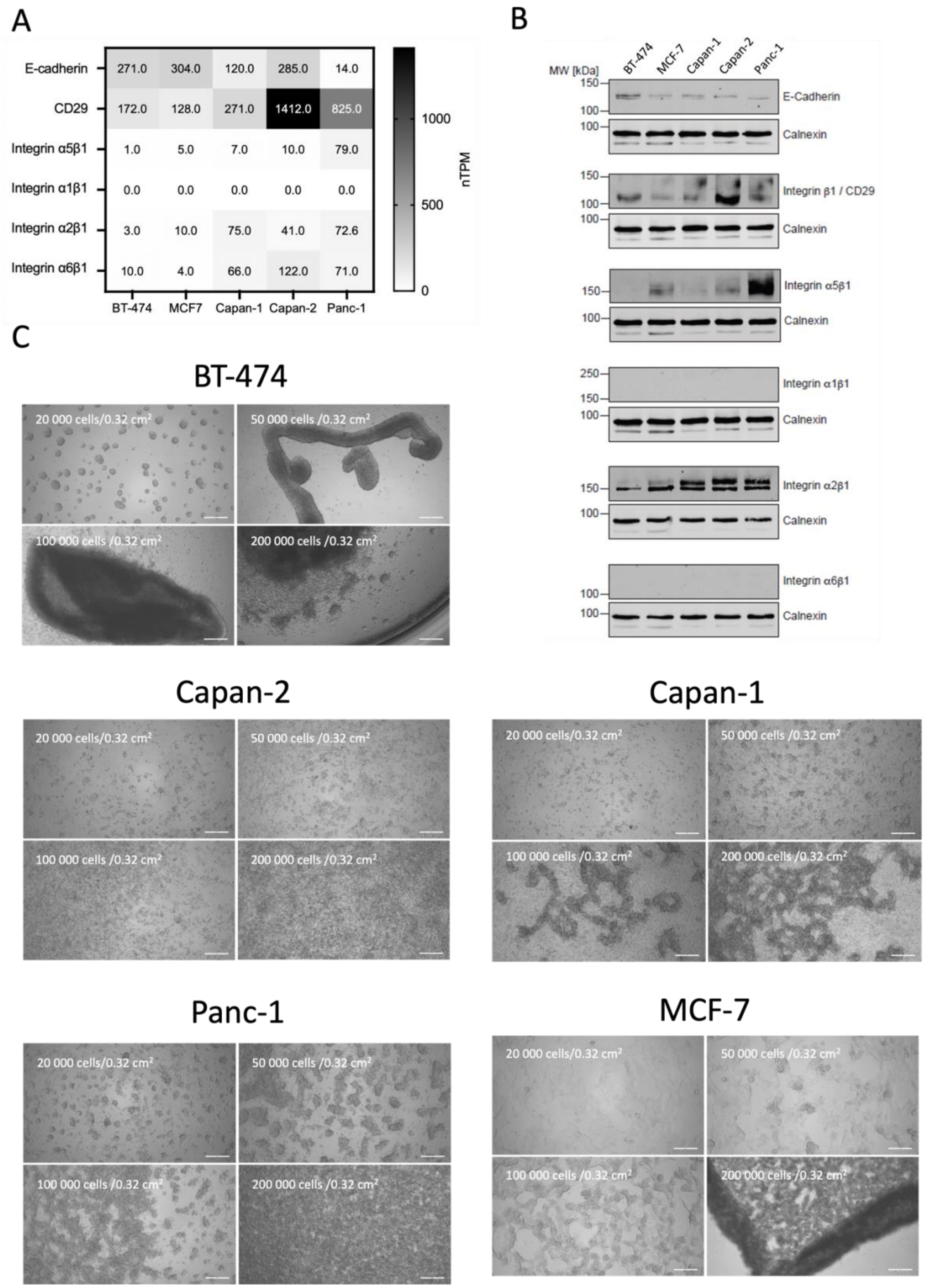

Figure S1: 3D cell culture cell lines. **A**) Normalized transcripts per million data for selected intercellular (cadherin) and extracellular (integrins) adhesion proteins in of the cell lines used in this study. Data was derived from the human protein atlas (proteinatlas.org). **B)** Western blot analysis of cell line lysates using antibodies specific for the selection of inter- and

extracellular adhesion proteins as indicated. Anti-Calnexin antibodies were used as loading control. Representative example from two biological replicates. **C)** Bright field microscopy data of the five cell lines used in this study, plated a various densities on low adhesive surfaces to induce 3D culture formation. Scale bars are 100 µm.

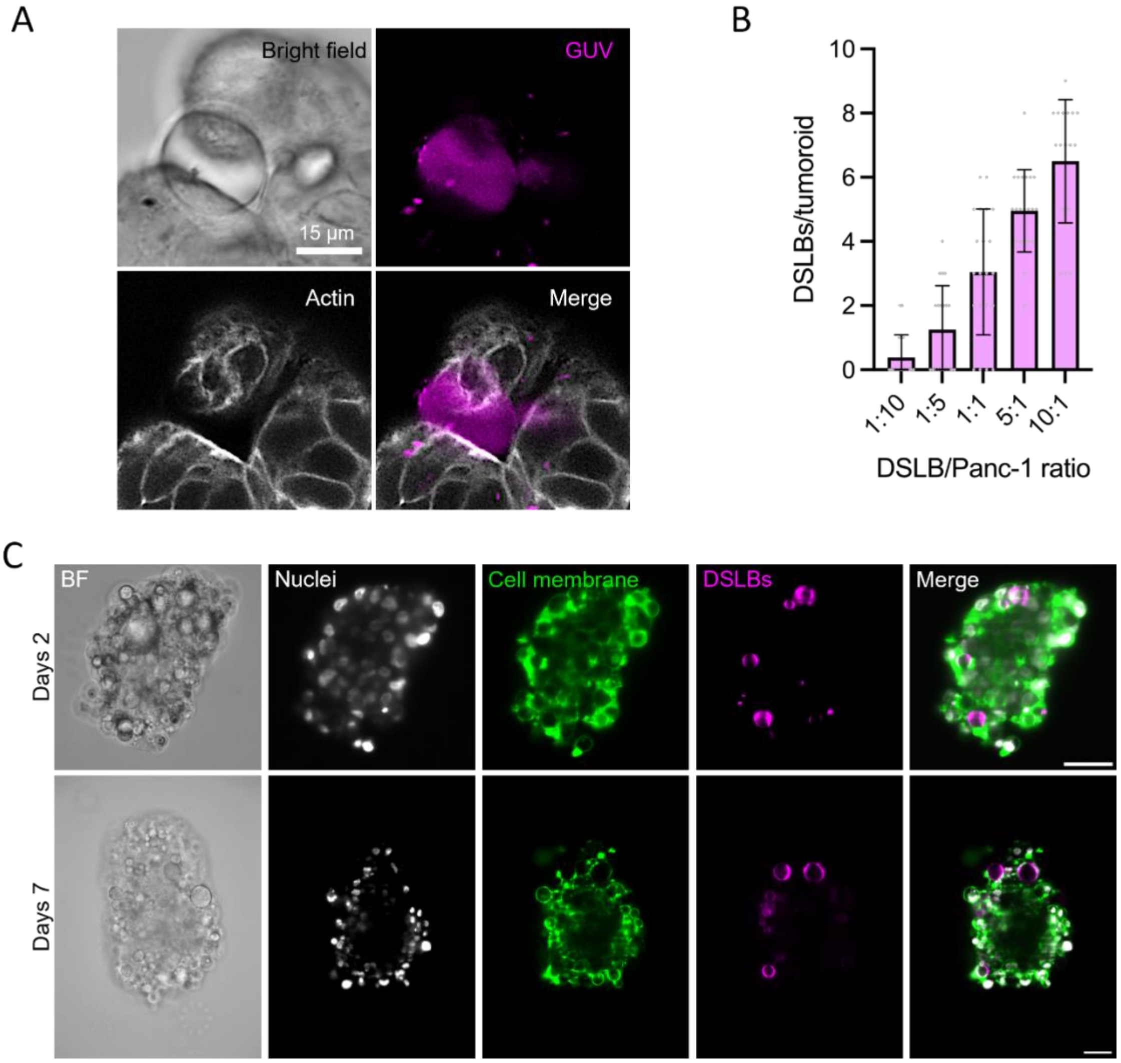

Figure S2: Stable integration of synthetic cells into tumoroids. **A)** Representative confocal microscopy maximal z-projections of Panc-1 tumoroid integrating a GUV as a synthetic cell model after 48 hours of culture. **B)** Quantification of DSLB-based synthetic cells integration into 3D cultures formed from Panc-1 cells after 48 hours of co-culture in various synthetic cell to Panc-1 ratios. Results shown as mean ±SD from n>21. **C)** Representative light sheet microscopy images of DSLB-based synthetic cells integrated into Panc-1 tumoroids 2 days and 7 days after formation. Cells were stained for membrane (wheat germ agglutinin, green) and nuclei (Hoechst, grey). Scale bar is 50 µm.

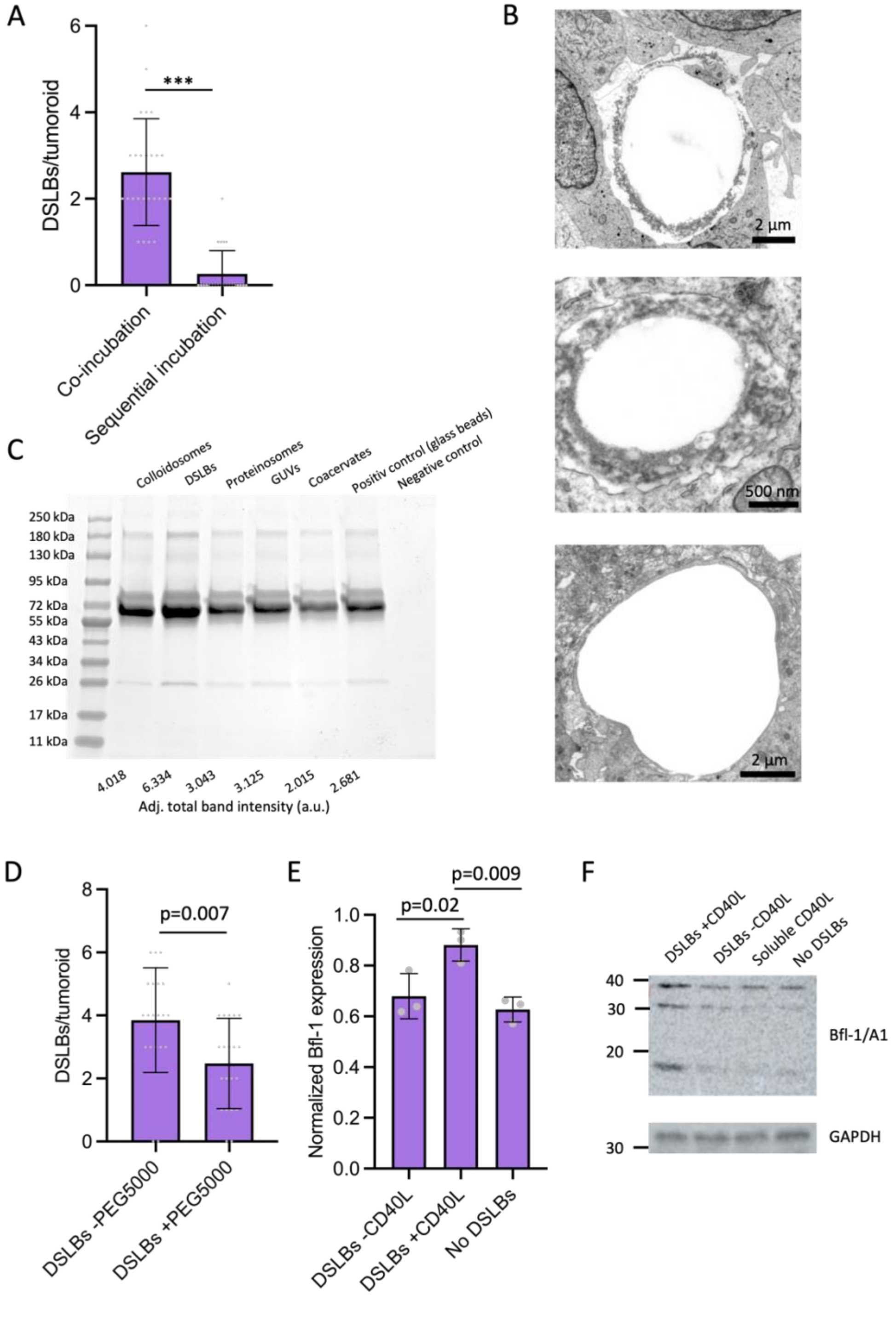

Figure S3: Self-assembly mechanisms of hybrid tumoroids. **A)** Quantification of DSLB-based synthetic cells integration into tumoroids formed from Panc-1 cells. DSLBs were either incubated with singularized synthetic cells for 48 hours (co-incubation), or added to pre-formed spheroids (sequential incubation). Results shown as mean ±SD from n>23, *** p<0.001 unpaired two-tailed t-test. **B)** Additional representative examples of the synthetic cell-cancer cell interface from TEM images, showing either the close contact between the synthetic cell membrane and the cell membrane or denser layers of surrounding ECM. **C)** SDS PAGE of serum opsonization levels from the synthetic cell panel incubated in 10% fetal bovine serum for 1 hour. Total band intensity for each lane is indicated in the bottom. **D)**

Quantification of DSLB-based synthetic cells integration into tumoroids formed from Panc-1 cells either with plain DSLBs or DSLB produced with 10 mol% PEG5000 on the lipid bilayer surface. Results shown as mean ±SD from n>20, p values as indicated from unpaired two-tailed t-test. **E)** Quantitative PCR of Bfl-1 RNA levels in Panc-1 hybrid tumoroids formed with DSLBs presenting CD40L, plain DSLBs or no DSLBs. Results are shown from 3 separate replicates shown as mean ±SD, normalized to RNA levels of glyceraldeyde-3-phosphate dehydrogenase. P values as indicated from one-way ANOVA. **F)** Western blot analysis of Bfl-1 expression in Panc-1 hybrid tumoroids after culture with DSLBs presenting CD40L, with plain DSLBs, with plain DSLBs and 1 µg/mL soluble CD40L and DSLB free cultures. GAPDH was used as loading control.

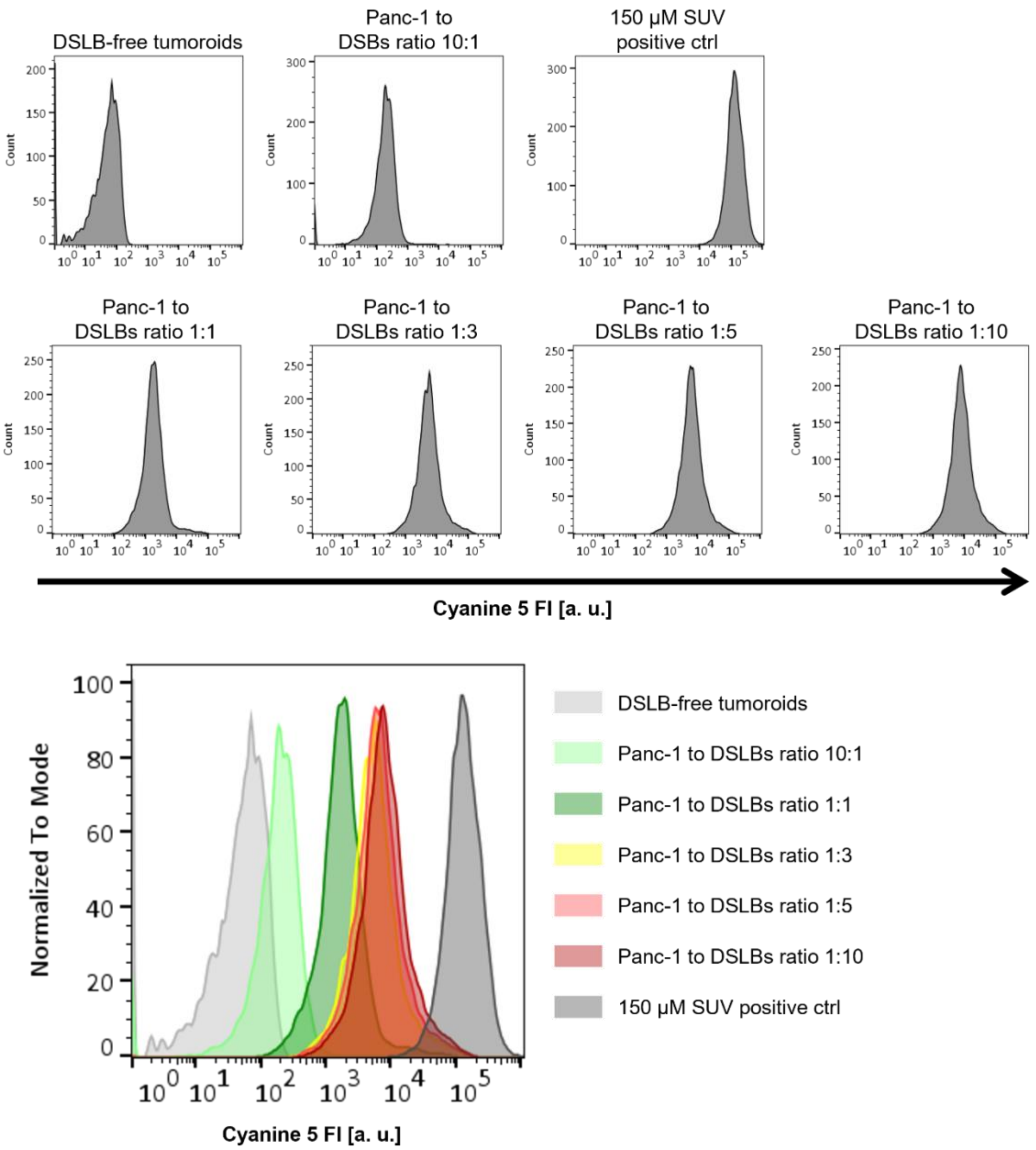

Figure S4: Contact events and membrane exchange between the Panc-1 cells and synthetic cells. Quantification of DSLB-membrane fluorescence intensity in Panc-1 cells of various hybrid tumoroid cultures. DSLBs featuring a membrane with a strong Cyanine 5 fluorescent signal were generated and used in the formation of hybrid tumoroids. Each condition was generated using 50 000 Panc-1 cells and varying numbers of DSLBs indicated by the Panc-1 to DSLBs ratio. After 48 hours of culture, the hybrid tumoroids were disassembled via trypsinization and subsequently fixed and stained for nuclei using Hoechst. Cyanine 5 fluorescent intensity in individual Panc-1 cells was analyzed via flow cytometry. DSLB-free tumoroids served as negative control and DSLB-free tumoroids cultivated for 48 hours in medium supplemented with 150 µM of the SUV stock solution utilized in DSLB generation were utilized as positive control.

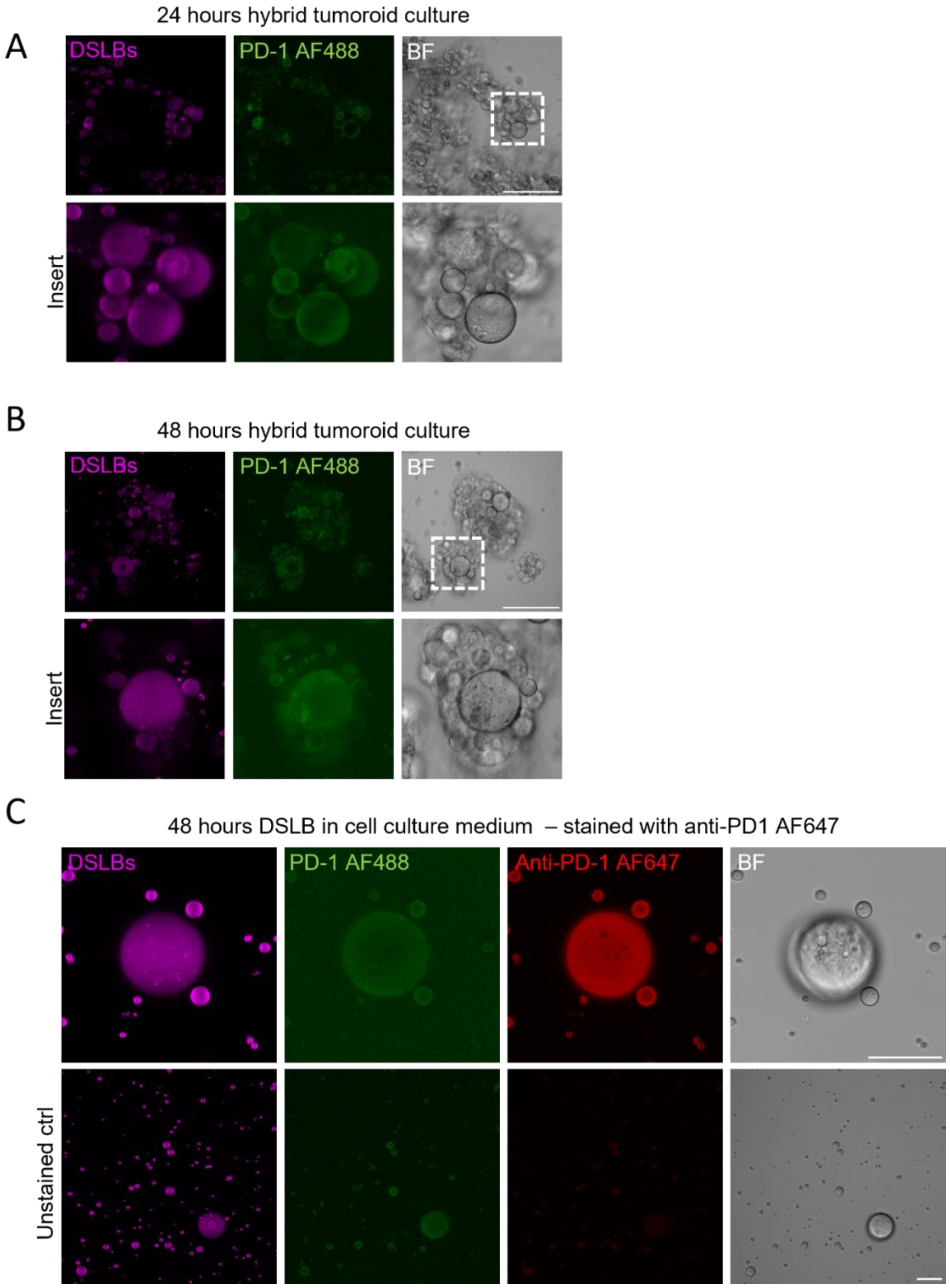

Figure S5: Stability of DSLB-protein functionalization. **A**) & **B**) Representative confocal microscopy maximal z-projections of tumoroids after 24 hours or 48 hours of culture. DSLBs were decorated with Alexa Fluor 488-labelled PD-1 and used in the formation of hybrid tumoroids. Scale bars are 150 µm. **C**) Enlarged confocal microscopy maximal z-projection of a DSLB decorated with fluorescent PD-1 after 48 hours of incubation in cell culture medium at 37°C. The stability of the protein was tested via staining with a specific anti-PD-1 antibody. Scale bars are 50 µm.

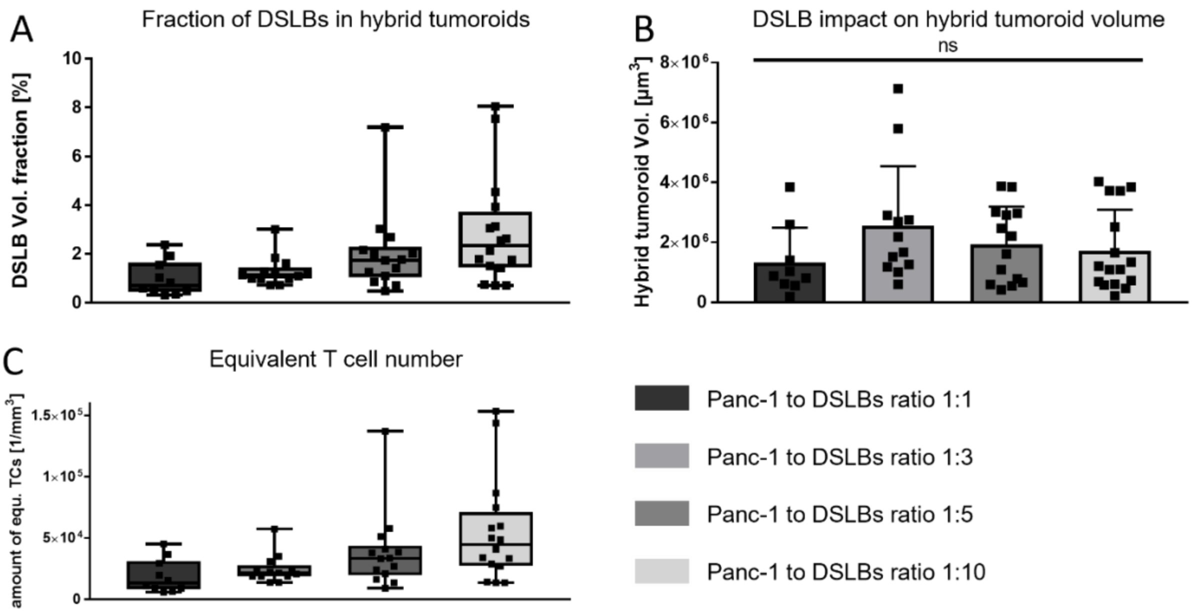

Figure S6: Impact of DSLB quantity used in hybrid tumoroid formation. **A)** Quantification of the volume fraction of a tumoroid consisting of integrated DSLB-based synthetic cells. Tumoroids were formed from 50 000 Panc-1 cells and varying numbers of DSLBs indicated by the Panc-1 to DSLBs ratio. Results are shown as a box plot comprised of min and max whiskers, all data points, upper and lower quartiles and median from n>10. **B)** Quantification of the total volume of hybrid tumoroids composed of Panc-1 cells and integrated DSLB-based synthetic cells. Results shown as mean +SD from n>10, ns p>0.05 one-way ANOVA. **C)** Quantification of the number of T cells equivalent to the density of integrated DSLB-based synthetic cells in various hybrid tumoroids. The total DSLB volume per tumoroid was used to calculate the number of T cells and normalized to a theoretical tissue volume of 1 mm^3. A T cell was estimated as a spherical object with a diameter of 10 µm. Results are shown as a box plot comprised of min and max whiskers, all data points, upper and lower quartiles and median from n>10.

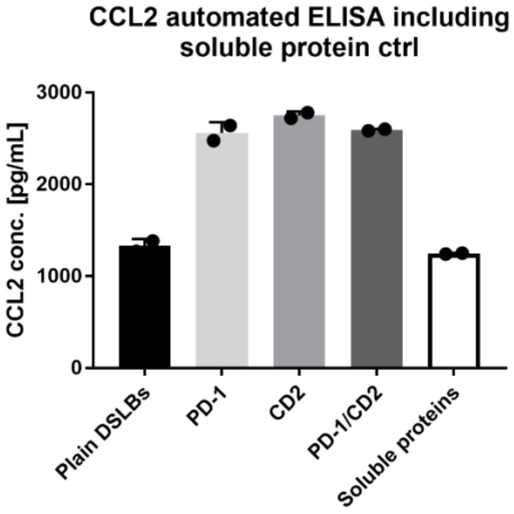

Figure S7: Enzyme-linked immunosorbent assay-based quantification of soluble CCL2 secreted from Panc-1 tumoroids with ART-TIMEs of varying composition after 48 hours of incubation. A soluble protein control was generated to rule out the impact of unbound protein left over from DSLB-functionalization. The concentration of soluble PD1 and CD2 was adjusted to 1.5 nM, reflecting the concentration range generated by ART-TIMES in Panc-1 tumoroids. Results shown as mean ±SD from 2 biological replicates measured in technical triplicates.

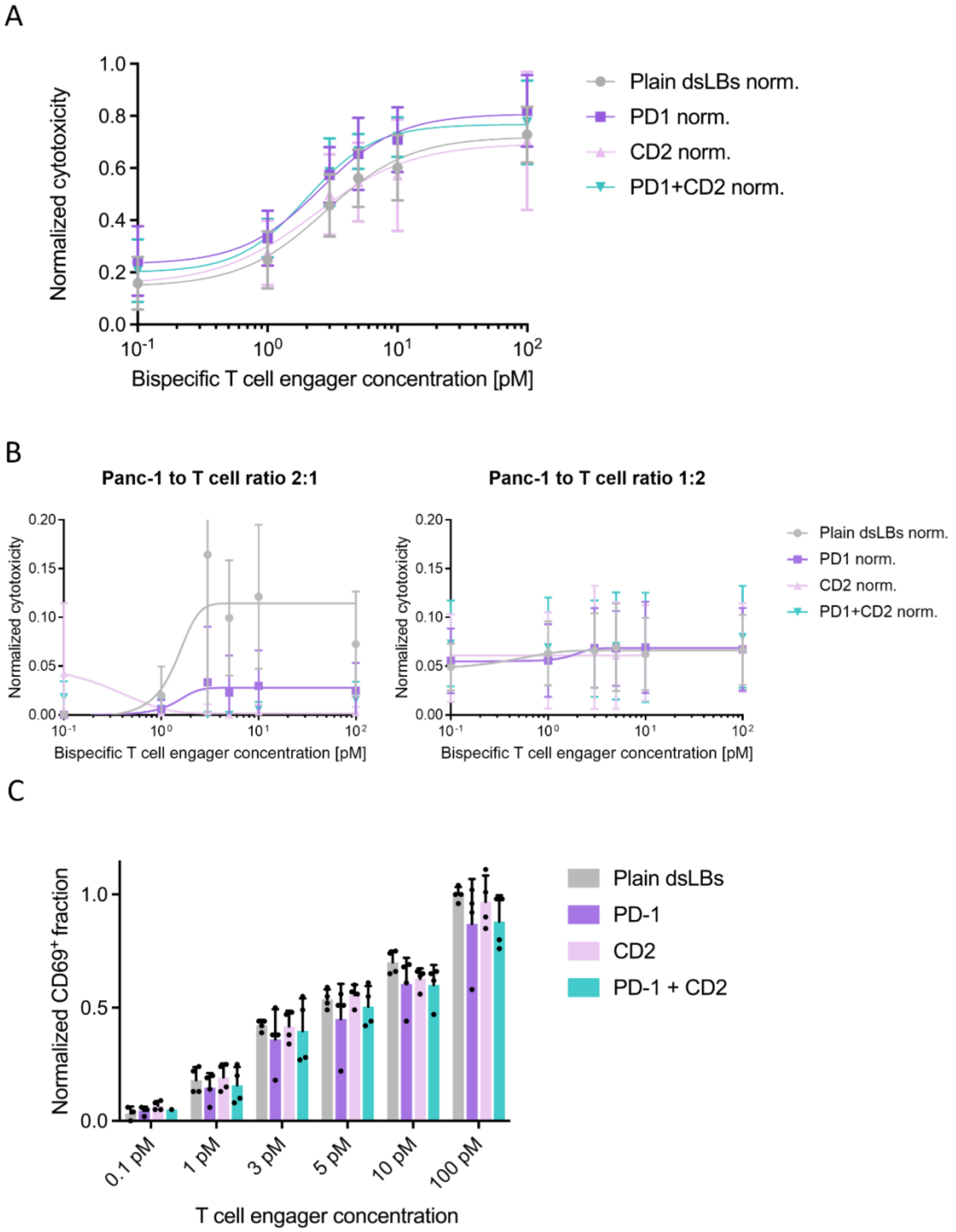

Figure S8 Bispecific T cell engager-mediated killing of hybrid tumoroids. **A)** Lactate dehydrogenase release assay for quantification of killing of ART-TIME containing Panc-1 hybrid spheroids by primary human CD8 T cells in a bispecific T cell engager dilution series. T cells were incubated with hybrid tumoroids preformed for 48 hours at a 5:1 T cell to cancer cell ratio. Results are shown from n=2 donors as mean ±SD from two replicates each. **B)** Lactate dehydrogenase release assay for quantification of killing of ART-TIME containing Panc-1 hybrid spheroids by primary human CD8 T cells in a bispecific T cell engager dilution series under suboptimal conditions. T cells were incubated with hybrid tumoroids preformed for 48 hours at a 2:1 or 1:2 T cell to cancer cell ratio. Results are shown from n=2 donors as mean ±SD from two replicates each. **C)** Flow cytometry quantification of the fraction of CD69

expressing primary human CD8 T cells incubated with ART-TIME containing Panc-1 3D cultures for 12 hours in the presence of increasing bispecific T cell engager concentrations. Results are shown from n=2 donors as mean $\pm$SD from two replicates each.

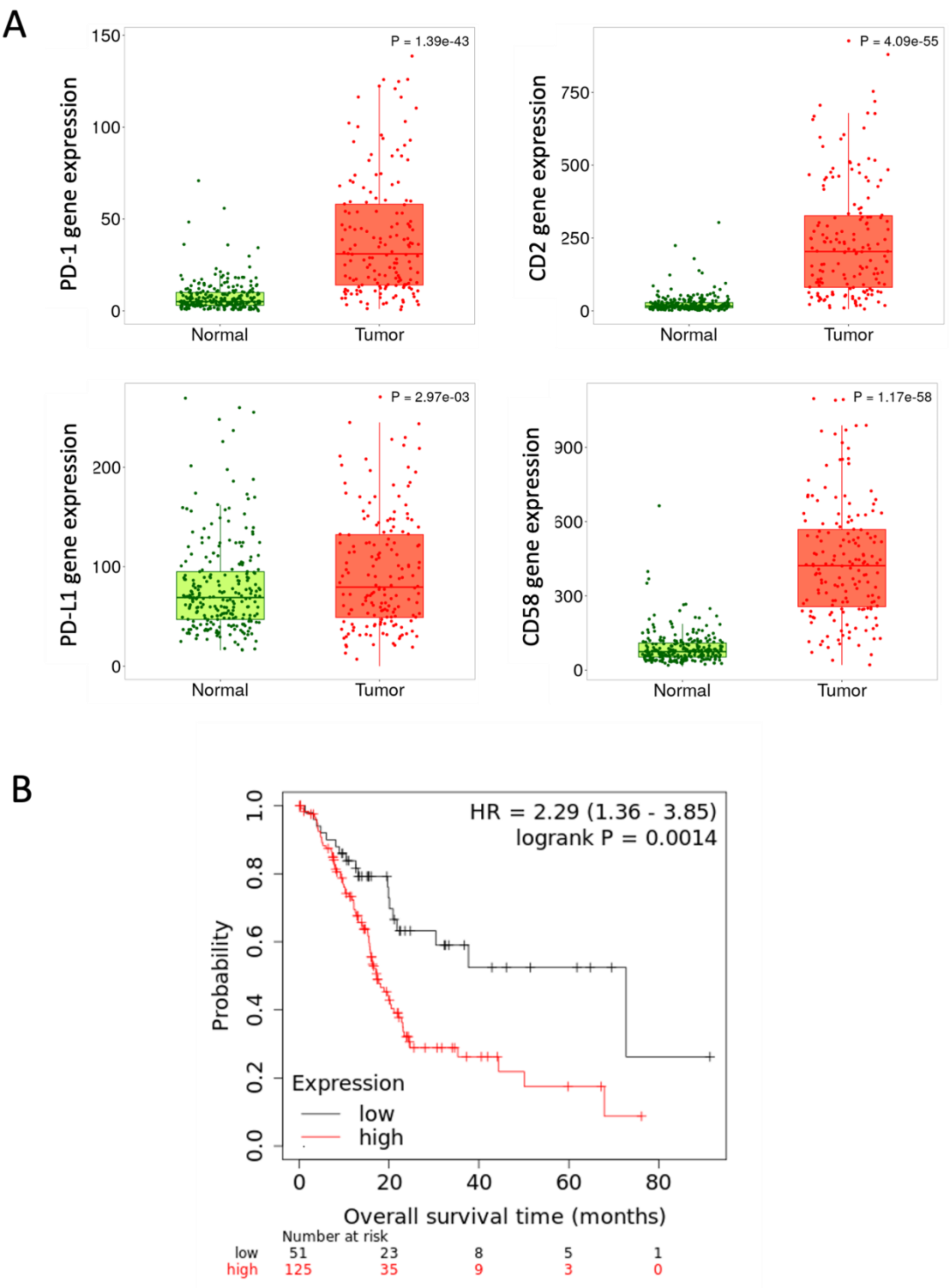

Figure S9 Clinical relevance of PD-1/PD-L1 and CD2/CD58 expression. RNA expression levels of PD-1 and PD-L1 as well as CD58 and CD2 from 177 patients samples quantified from tumorous tissue and 252 normal pancreatic tissues. Results are shown as a box plot comprised of min and max whiskers, all data points and outliers, upper and lower quartiles and median. **B)** Survival analysis in pancreatic adenocarcinoma samples using the combined expression of PDL1 and CD58 and overall survival data shows markedly worse outcome in those with higher expression.

Supplementary note 1:

Dispersed liquid-liquid phase-separated droplet-supported lipid bilayers (dsLBs) are a class of synthetic cell structures based on micrometer-sized oil droplets, stabilized by anionic surfactants and coated with phospholipid bilayers. They are formed using an oil-in-water (o/w) emulsion method where polydimethylsiloxane (PDMS) droplets are produced, stabilized by surfactants like sodium dodecyl sulfate (SDS), and subsequently coated with small unilamellar vesicles (SUVs) using charge-mediated assembly (see supplementary note 1 figure).

The assembly process begins with emulsification of PDMS in the presence of SDS, followed by the addition of divalent cations (e.g., Mg^{2+}) to facilitate the attachment of net-negative charged SUVs, which are composed of functionalized lipids. This results in a stable lipid bilayer coating on the droplet surfaces. The SUVs typically include lipids like EggPC, PE-DGS-NTA(Ni^{2+}) for protein attachment, and fluorescent lipids for visualization. The dsLB technology allows for the production of ultra-soft substrates (~1 kPa) with the ability to modulate stiffness by cross-linking the PDMS core, extending into the MPa range. This is achieved by curing the PDMS droplets with a cross-linking agent. These dsLBs can also be functionalized with proteins, such as through histidine-tagged proteins with NTA-functionalized lipids. This approach allows for controlled presentation of molecules and ligands, which typically transmit intercellular signals.

The advantages of dsLBs include lateral mobility of ligands, which promotes a more efficient formation of signalosomes at intercellular interfaces and improved mechano-mimetic properties based on their soft oil core. Overall, dsLBs offer a versatile platform for biophysical modulation and protein functionalization, enabling the study of membrane dynamics, ligand mobility, and stiffness in a highly controlled and tunable environment.

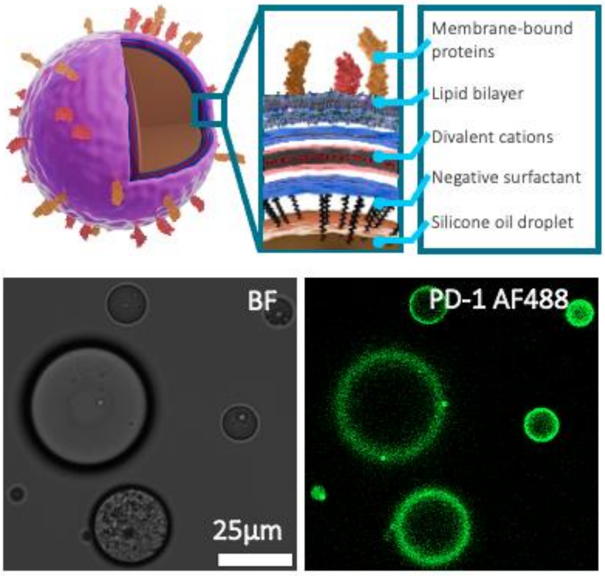

Supplementary note 1 figure: Schematic illustration of a dsLB interface (top panel) together with bright field and fluorescence microscopy images of dsLB functionalized with AlexaFluor488 labeled recombinant human PD-1 (bottom panel).
