# Supplementary material for: Self-assembly of hybrid 3D cultures by integrating living and synthetic cells
Source: Nat Commun. 2025 Dec 10;16:11073. doi: 10.1038/s41467-025-66789-3 (PMC12698700; doi:10.1038/s41467-025-66789-3)
Supplement: Supplementary file 2 — Description of Additional Supplementary Files [file 41467_2025_66789_MOESM2_ESM.pdf]

Description of Additional Supplementary Files

Supplementary Movie 1: Live cells time laps imaging of dispersed Panc-1 cells (brigth field) cultured with DSLBbased synthetic cells (magenta).

Supplementary Movie 2: Live-cells time laps imaging of Free floating Panc-1 tumoroid hybird with integrated DSLB synthetic cells (left) and one representative manually segmented synthetic cells contour (right).
