# Supplementary material for: Self-assembly of hybrid 3D cultures by integrating living and synthetic cells
Source: Nat Commun. 2025 Dec 10;16:11073. doi: 10.1038/s41467-025-66789-3 (PMC12698700; doi:10.1038/s41467-025-66789-3)
Supplement: Supplementary file 6 — Transparent Peer Review file [file 41467_2025_66789_MOESM6_ESM.pdf]

nature portfolio

Peer Review File

Self-assembly of hybrid 3D cultures by integrating living and synthetic cells

Corresponding Author: Dr Oskar Staufer

This file contains all reviewer reports in order by version, followed by all author rebuttals in order by version.

Version 0:

Reviewer comments:

Reviewer #1

(Remarks to the Author)
In this contribution, the authors report on the stable integration of artificial cells in a 3D tumoroid. They demonstrate that integration takes place during the growth phase of the 3D tissue and that opsonization plays an important role in the success of uptake. They furthermore investigate if the interplay between artificial and living cells can be used in immune modulation. This is an ambitious investigation, which in fact contains two different concepts: the integration study and the cell response. Both could be the basis for a separate paper. The combination now leads to some additional significant questions that need to be answered to make the paper suitable for publication.
With respect to the artificial cells. The authors show that opsonization is important, but also mention that other parameters could play a role. I think that size and mechanical properties of the artificial cell should be studied in more detail. Smaller particles will potentially be phagocytosed rather than integrated and it is important to establish this relationship. For example, is the picture of Fig 2B not an attempt to phagocytosis? I would expect GUVs to be mechanically too fragile to withstand incorporation in the tumoroid structure. The authors should investigate this.
Regarding the biological evaluation. My main concern is the dynamic character of the NiNTA-His tag interaction. This noncovalent interaction is not stable in cell culture medium, which would lead to loss of the ligands from the surface. The authors should first quantify the amount of ligands introduced on the artificial cells, and also demonstrate, with e.g. fluorescently labeled species their fate after integration in the tumoroid. The authors should also discuss their availability after opsonization.
With respect to the biological assays, the authors should include the controls in Figure 3 where both PD1 and CD2 are added in solution (without DSLBs) to clearly demonstrate a scaffolding effect. Also DCD40L should be added in solution as a control in Fig 2. Furthermore, since only a fraction of the cancer cells is in contact with the artificial ones, I would expect a heterogeneous response of the tumoroid. The authors should try to analyze if there are two populations present.
The biological outcome is not very consistent. There is no real pattern to be observed from the effect of PD1, CD2 or the combination in Fig 3E. How can these results be explained? Based on the large variability, I am not sure if any biological conclusion can be drawn.
Minor comments
Fig 1 C Could the authors indicate how many living cells are in the tumoroid; what is the ratio between artificial and living cells after the tumoroid has been fully formed?
I can’t find movie S2.
Line 15 page 10 fig 3F should be 2F

Reviewer #2

(Remarks to the Author)
In this manuscript, the authors present an innovative approach to generate 3D cancer cell cultures by systematically evaluating different synthetic cell mimetics as scaffolds/inducers of 3D culture formation. They assess the underlying principles and utilize this novel model system to study tumor receptor co-signaling. The study reveals new insights into tumor immunosuppression via PD1 and CD2, provides relevant data using a bi-specific T cell engager, and correlates the signature with overall survival in pancreatic carcinoma patients. This well-written manuscript demonstrates significant innovation and potential for studying cancer-immune interactions in 3D cell cultures. However, several points should be

addressed to strengthen the significance of their findings:

1. While the authors establish the importance of adhesion molecules in 3D culture formation, their data relies solely on transcriptomic analyses. Protein expression data for E-cadherin, CD29, and key expressed integrins would provide valuable validation.
2. The authors should address the long-term stability of their model system, including maximum culture duration and whether prolonged culture affects architecture and/or cellular composition.
3. The CD40 experiment demonstrates Bfl1 transcription but not protein expression. Given that transcription and translation don't always correlate directly, analyzing Bfl1 protein expression would strengthen these findings.
4. Additional replicates for the experiments shown in Figures 3J and 3K would enhance statistical significance.
5. While the authors note differences in T cell activation between groups (plain DSLC, CD2, PD-1, CD2/PD-1) but no statistical difference in killing potency, their analysis uses only one T cell:target ratio (5:1). Testing multiple ratios, particularly sub-optimal ones, would provide more comprehensive insights.

Version 1:

Reviewer comments:

Reviewer #1

(Remarks to the Author)
The authors have provided novel data and clear answers to the questions raised. Based on this additional information I feel the paper is now suited for publication

Reviewer #2

(Remarks to the Author)
The authors have adequately addressed all concerns raised in the previous review. I have no additional comments or issues to be addressed.

Open Access This Peer Review File is licensed under a Creative Commons Attribution 4.0 International License, which permits use, sharing, adaptation, distribution and reproduction in any medium or format, as long as you give appropriate credit to the original author(s) and the source, provide a link to the Creative Commons license, and indicate if changes were made.
In cases where reviewers are anonymous, credit should be given to 'Anonymous Referee' and the source.
The images or other third party material in this Peer Review File are included in the article’s Creative Commons license, unless indicated otherwise in a credit line to the material. If material is not included in the article’s Creative Commons license and your intended use is not permitted by statutory regulation or exceeds the permitted use, you will need to obtain permission directly from the copyright holder.
To view a copy of this license, visit https://creativecommons.org/licenses/by/4.0/

REVIEWER COMMENTS

Reviewer #1:

In this contribution, the authors report on the stable integration of artificial cells in a 3D tumoroid. They demonstrate that integration takes place during the growth phase of the 3D tissue and that opsonization plays an important role in the success of uptake. They furthermore investigate if the interplay between artificial and living cells can be used in immune modulation.
This is an ambitious investigation, which in fact contains two different concepts: the integration study and the cell response. Both could be the basis for a separate paper. The combination now leads to some additional significant questions that need to be answered to make the paper suitable for publication.

We thank the reviewer for his/her careful assessment and the appreciation that our study represents an interdisciplinary approach unifying two concepts. We would like to address the suggestions for further improvement in the following point-by-point response.

Point1: *With respect to the artificial cells. The authors show that opsonization is important, but also mention that other parameters could play a role. I think that size and mechanical properties of the artificial cell should be studied in more detail. Smaller particles will potentially be phagocytosed rather than integrated and it is important to establish this relationship. For example, is the picture of Fig 2B not an attempt to phagocytosis?*

The reviewer brings up two highly relevant points for our study. First, we very much agree that the mechanical properties of the synthetic cells are crucial for their stable integration into the tumoroid. We have previously reported the Young´s modulus of silica colloidosomes (212.3 kPa ± 161.0 kPa, Hakami *et al. Adv. Health. Mat*., 2024) and DSLBs (3.85 kPa ±1.60 kPa, kPa, Burgstaller *et al. Small*, 2024), while a range of elastic moduli has been reported for GUVs (e.g. 5 kPa, Campillo *et al. Mat. Sci. and Eng. C*, 2009) proteinosomes (e.g. 27 kPa,Huang *et al. Nat. Comm.* 2013) and coacervates (e.g. 0.43 kPa, *Novosedlik et al. Nat. Chem.* 2025). While they do not correlate with the integration efficacy, the deformability of our synthetic cells is most likely key to sustain appropriate receptor-ligand signaling at the living-synthetic cell interface (see Fig. 2D,E,F and Movie S2).
In the revised version of our manuscript, we now write on page 9, line 22 to page 10, line 2: "*Importantly, the interactions between natural and synthetic cells were not merely transient but persisted post-integration. Time-resolved segmentation of individual synthetic cells within tumoroids from live cell imaging data revealed that the elastic DSLBs, which have comparable stiffness to natural cells (~1 kPa)[13], underwent constant deformation within the tumoroids (**Fig. 2D,E,F** and **Movie S2**).*"

Second, the relevance of size distribution of the synthetic cells and their internalization by the living cells. While this aspect is mostly dependent on the endocytotic activity of the living cells (e.g. fibroblasts have higher uptake rates then epithelial cells), smaller particles tend to undergo fast intracellular uptake, a process that we have studied in detail for GUVs and cell cultures (Staufer *et al. Biomaterials,* 2021). Based on this reviewer's comment, we now performed a detailed investigation of the size distribution and uptake, specifically of DSLBs, within the hybrid tumoroid. For this, we performed 3D light sheet microscopy and 3D

rendering to measure sizes and volume fraction of the DSLBs within the 3D cultures. By comparing size distribution between the initial DSLB population and those within the tumoroids, we found that the tumoroids preferentially integrate larger synthetic cells with an average size of 9.4 µm and mean volume fraction between 0.8-2.2 (see also reviewer 1 point 5). Moreover, our imaging revealed that the living cells do engulf the synthetic cells in certain instances, with fractions of the synthetic cell membrane (visualized by the fluorescent lipids) taken up into the intracellular space. However, these fractions did not exhibit the high refractive index of the oil core of the DSLBs and were reminiscent of trogocytosis events in which the membrane of the DSLBs was endocytosed (this observation was later applied for the investigation of point 4 under reviewer 1).
In the revised version of our manuscript, we now write on page 10, lines 3-11: "*Importantly, we also found evidence of trogocytosis-like behavior in which the cancer cells internalized small parts of the DSLB fluorescent membrane (**Fig. S4A**). By disintegrating the hybrid tumoroids after culture and quantifying the fluorescent membrane content in the cancer cells via flow cytometry, we observed that at a ratio of 1:10 cancer cells to synthetic cells, every cancer cell integrated some level of membrane fluorescence during the 48 hour culture period (**Fig. S4B**). The corresponding broad distribution of contacts indicates that under these conditions hybrid tumoroid assembly is highly dynamic and involves continuous remodeling. In contrast, not every cancer cell showed membrane fluorescence at ratios below 1:3.*"

With regards to Figure 2B, as TEM imaging is performed on a thin 2D slice of the sample, major conclusions about the degree of internalization cannot be drawn from this. Importantly, during our TEM analysis, we did not see any indication of significant phagocytosis but several examples of close contact formation of the cell membrane to the synthetic cells or formation of protrusions to the synthetic cell surface through a surrounding ECM scaffold. However, we agree with the reviewer that this is not obvious from the figure presented in 2B and therefore now include additional examples in Figure S3B. From this we conclude that synthetic cells are not internalized into the living cells, at least to a high efficacy, but parts of their membrane are. This is an important process in the tumor immune microenvironment and further underscores the degree of mimicry in our synthetic approach.

The new results referred to above were now integrated as Figures 2D,E,F; S3B; S4 and S6:

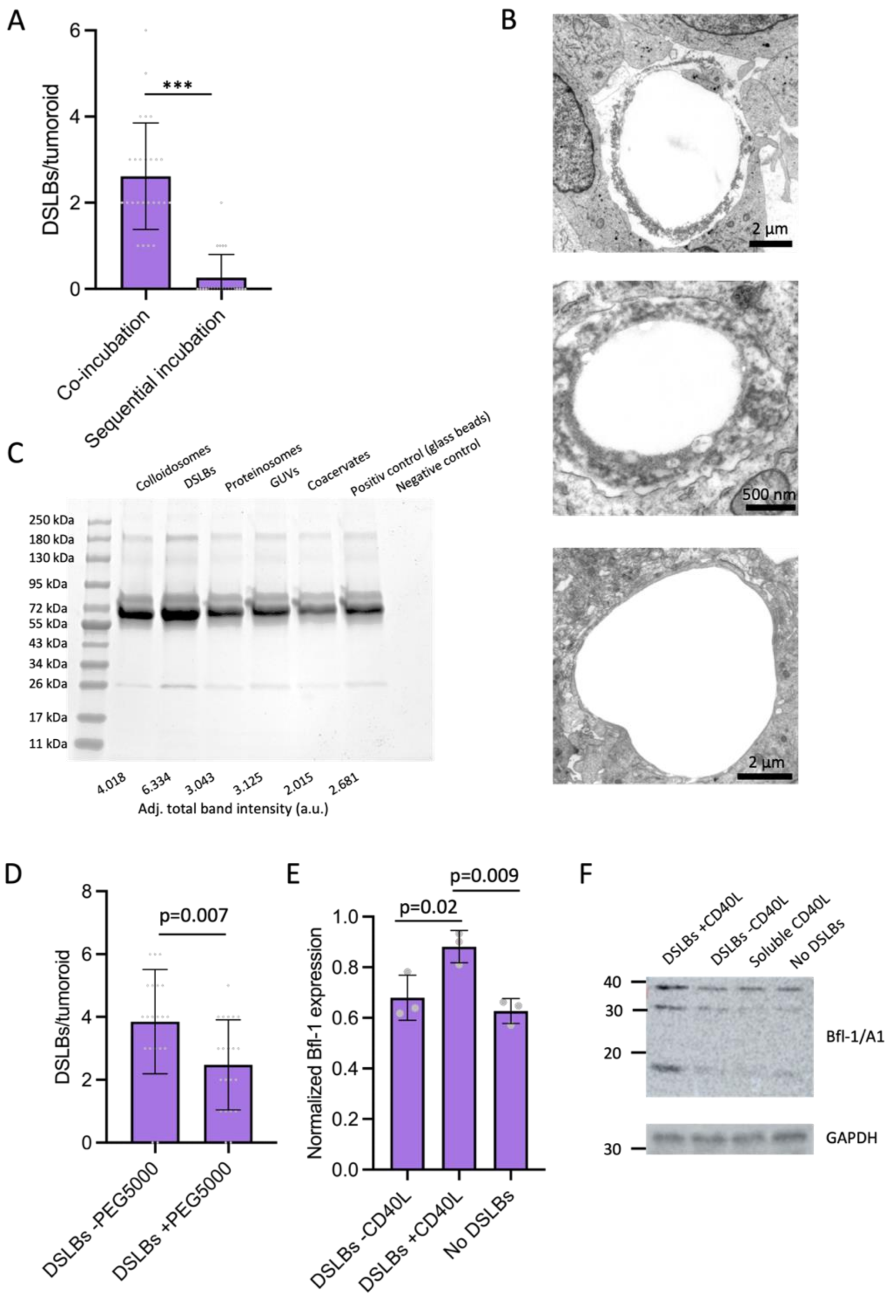

Figure S3: Self-assembly mechanisms of hybrid tumoroids. **A)** Quantification of DSLB-based synthetic cells integration into tumoroids formed from Panc-1 cells. DSLBs were either incubated with singularized synthetic cells for 48 hours (co-incubation), or added to pre-formed spheroids (sequential incubation). Results shown as mean ±SD from n>23, *** p<0.001 unpaired two-tailed t-test. **B)** Additional representative examples of the synthetic cell-cancer cell interface from TEM images, showing either the close contact between the synthetic cell membrane and the cell membrane or denser layers of surrounding ECM. **C)** SDS PAGE of serum opsonization levels from the synthetic cell panel incubated in 10% fetal bovine serum for 1 hour. Total band intensity for each lane is indicated in the bottom. **D)** Quantification of DSLB-based synthetic cells integration into tumoroids formed from Panc-1 cells either with plain DSLBs or DSLB produced with 10 mol% PEG5000 on the lipid bilayer surface. Results shown as mean ±SD from n>20, p values as indicated from unpaired two-tailed t-test. **E)** Quantitative PCR of Bfl-1 RNA levels in Panc-1 hybrid tumoroids formed with DSLBs presenting CD40L, plain DSLBs or no DSLBs. Results are shown from 3 separate replicates shown as mean ±SD, normalized to RNA levels of glyceraldeyde-3-phosphate dehydrogenase. P values as indicated from one-way ANOVA. **F)** Western blot analysis of Bfl-1 expression in Panc-1 hybrid tumoroids after culture with DSLBs presenting CD40L, with plain DSLBs, with plain DSLBs and 1 µg/mL soluble CD40L and DSLB free cultures. GAPDH was used as loading control.

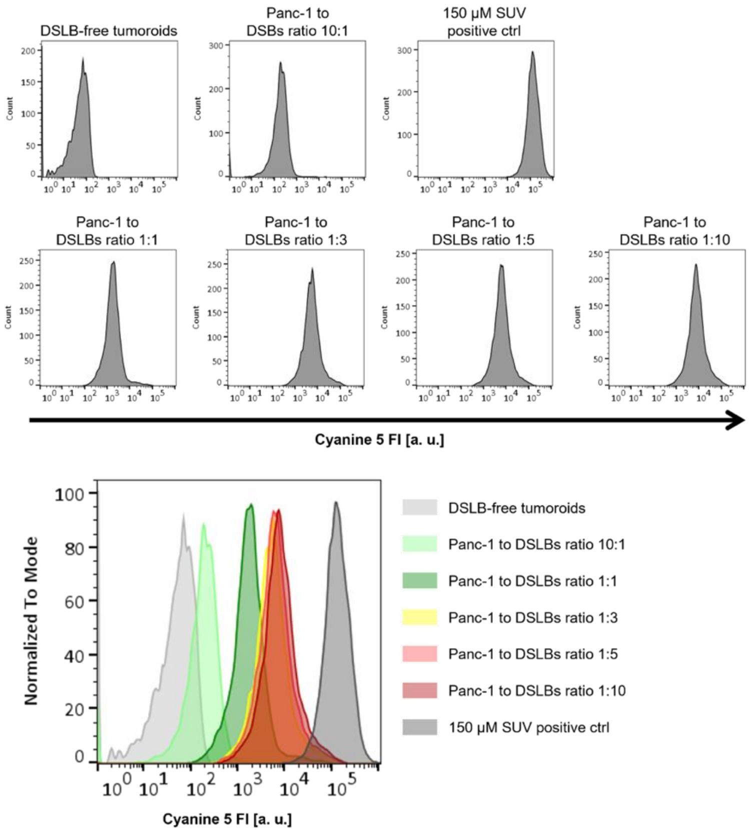

Figure S4: Contact events and membrane exchange between the Panc-1 cells and synthetic cells. Quantification of DSLB-membrane fluorescence intensity in Panc-1 cells of various hybrid tumoroid cultures. DSLBs featuring a membrane with a strong Cyanine 5 fluorescent signal were generated and used in the formation of hybrid tumoroids. Each condition was generated using 50 000 Panc-1 cells and varying numbers of DSLBs indicated by the Panc-1 to DSLBs ratio. After 48 hours of culture, the hybrid tumoroids were disassembled via trypsinization and subsequently fixed and stained for nuclei using Hoechst. Cyanine 5 fluorescent intensity in individual Panc-1 cells was analyzed via flow cytometry. DSLB-free tumoroids served as negative control and DSLB-free tumoroids cultivated for 48 hours in medium supplemented with 150 µM of the SUV stock solution utilized in DSLB generation were utilized as positive control.

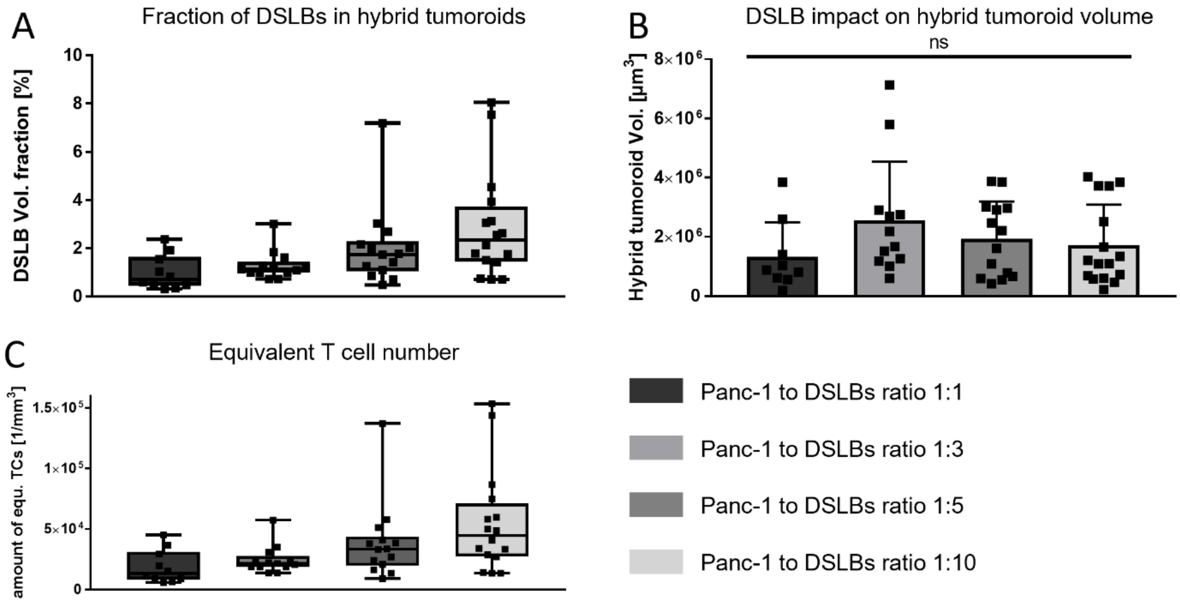

Figure S6: Impact of DSLB quantity used in hybrid tumoroid formation. **A)** Quantification of the volume fraction of a tumoroid consisting of integrated DSLB-based synthetic cells. Tumoroids were formed from 50 000 Panc-1 cells and varying numbers of DSLBs indicated by the Panc-1 to DSLBs ratio. Results are shown as a box plot comprised of min and max whiskers, all data points, upper and lower quartiles and median from n>10. **B)** Quantification of the total volume of hybrid tumoroids composed of Panc-1 cells and integrated DSLB-based synthetic cells. Results shown as mean +SD from n>10, ns p>0.05 one-way ANOVA. **C)** Quantification of the number of T cells equivalent to the density of integrated DSLB-based synthetic cells in various hybrid tumoroids. The total DSLB volume per tumoroid was used to calculate the number of T cells and normalized to a theoretical tissue volume of 1 mm^3. A T cell was estimated as a spherical object with a diameter of 10 µm. Results are shown as a box plot comprised of min and max whiskers, all data points, upper and lower quartiles and median from n>10.

Point 2: *I would expect GUVs to be mechanically too fragile to withstand incorporation in the tumoroid structure. The authors should investigate this.*

The reviewer mentions an interesting point concerning the GUVs applied in our study. GUVs feature an aqueous core and have a low elastic modulus (e.g. 5 kPa, Campillo *et al. Mat. Sci. and Eng. C*, 2009). As water can diffuse through their membrane, GUVs can be easily inflated or deflated by osmotic pressure and therefore tuned in their mechanical properties. The GUVs in our study were produced by a droplet-supported templating approach under iso-osmotic conditions, resulting in rigid inflated GUVs (Staufer *et al. Biomaterials*, 2021). This state persisted even after integration into the tumoroids, where we observed clearly intact GUVs even after 48 hours of culture.
In the revised version of our manuscript, we now write on page 5, lines 19-22: *"Of note, all integrated synthetic cell models remained structurally intact over the 48 hour culture period, including the more fragile GUVs, for which no ruptured vesicles could be observed (**Fig. S2A**). This indicates that the integration process is gentle and non-destructive for the synthetic cells."*

These results were now integrated as Figure S2A in the revised version of our manuscript:

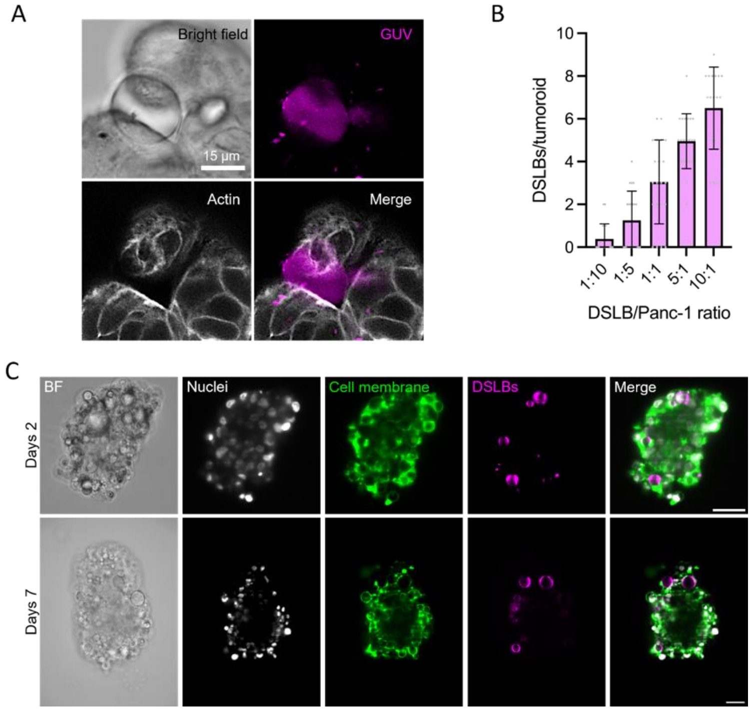

Figure S2: Stable integration of synthetic cells into tumoroids. **A)** Representative confocal microscopy maximal z-projections of Panc-1 tumoroid integrating a GUV as a synthetic cell model after 48 hours of culture. **B)** Quantification of DSLB-based synthetic cells integration into 3D cultures formed from Panc-1 cells after 48 hours of co-culture in various synthetic cell to Panc-1 ratios. Results shown as mean ±SD from n>21. **C)** Representative light sheet microscopy images of DSLB-based synthetic cells integrated into Panc-1 tumoroids 2 days and 7 days after formation. Cells were stained for membrane (wheat germ agglutinin, green) and nuclei (Hoechst, grey). Scale bar is 50 µm.

Point 3: *Regarding the biological evaluation. My main concern is the dynamic character of the NiNTA-His tag interaction. This noncovalent interaction is not stable in cell culture medium, which would lead to loss of the ligands from the surface. The authors should first quantify the amount of ligands introduced on the artificial cells, and also demonstrate, with e.g. fluorescently labeled species their fate after integration in the tumoroid. The authors should also discuss their availability after opsonization.*

The reviewer highlights a highly relevant point that we agree is fundamental for our approach and which we now evaluated in more detail. The ligand density applied on our DSLBs was 300 molecules/µm^2, determined by a protocol based on molar equivalents of soluble fluorophore (MESF) beads that we used for previous determination of ligand densities on membranes (see page 14, line 11 and page 26, line 24 as well as Burgstaller *et al. Small*, 2024 for the calibration procedure). There are conflicting reports on the stability of NTA(Ni^{2+})-His tag bonds under *in vivo* and cell culture conditions. While some reports highlight that the blood stability of NTA(Ni^{2+})-His tag bonds of recombinant proteins on vesicles is limited to 1-4 hours (Platt *et al. Bioconjug. Chem. 2011*), other *in vitro* approaches show that ligands on lipid membranes conjugated *via* NTA and incubated in 100% serum are stable with a half-life of approximately 4 days (Chen *et al. Bioconjug. Chem.* 2019). The exact determinants for stability in each system are not fully understood but mostly likely involve the density of NTA(Ni^{2+}) in the membrane, the length of the His tag and the stochiometric ratio between both. In order to validate the stability for our system, we coupled recombinant poly-histidine-tagged AlexaFluore488-conjugated PD-1 ectodomains to our DSLBs and formed hybrid tumoroids. Even after 48 hours of culture, we were able to image the PD-1-AF488 on the DSLB surface. The signal intensity was comparable to that of DSLBs kept in cell culture medium for the same time and to the initial signal intensity. Moreover, we could not detect intracellular PD-1-AlexaFluor488 signal above autofluorescence levels in the cancer cells. This demonstrates that the ligands coupled on the DSLB membrane remain presented in large parts over the complete culturing period. Moreover, we were able to stain the PD-1 on the synthetic cell membrane within the hybrid tumoroids with anti-PD-1 antibodies after 48 hours of culture. This indicates that the opsonization coat on the membrane is not dense/thick enough to prevent protein-protein interactions and the ligands remain accessible for receptor binding.

In the revised version of our manuscript, we now write on page 11, lines 2-13: *"We next verified that the protein coupled to the synthetic cell surface is presented to the cancer cells over the complete culture period. For this, we coupled a recombinant human PD-1 ectodomain labeled with AlexFluor488 on the DSLB surface and formed hybrid tumoroids (**Fig. S5A,B**). We found that PD-1 can be detected on the synthetic cell surface after 24 hours in culture and with only minimal reduced fluorescence after 48 hours. This was similar to the results obtained with DSLBs stored for 48 hours in culture media without cells (**Fig. S5C**), indicating that proteins coupled to the DSLB membrane via NTA(Ni^{2+}) remain presented to the cancer cells over the culture period. Moreover, we successfully stained the PD-1-presenting DSLBs within the hybrid*

*tumoroids with fluorescent anti-PD-1 antibody, indicating that despite the opsonization coat, conjugated receptor ligands remain accessible for binding (**Fig. S5C**).“*

These results have been added as Figure S5 our revised manuscript version.

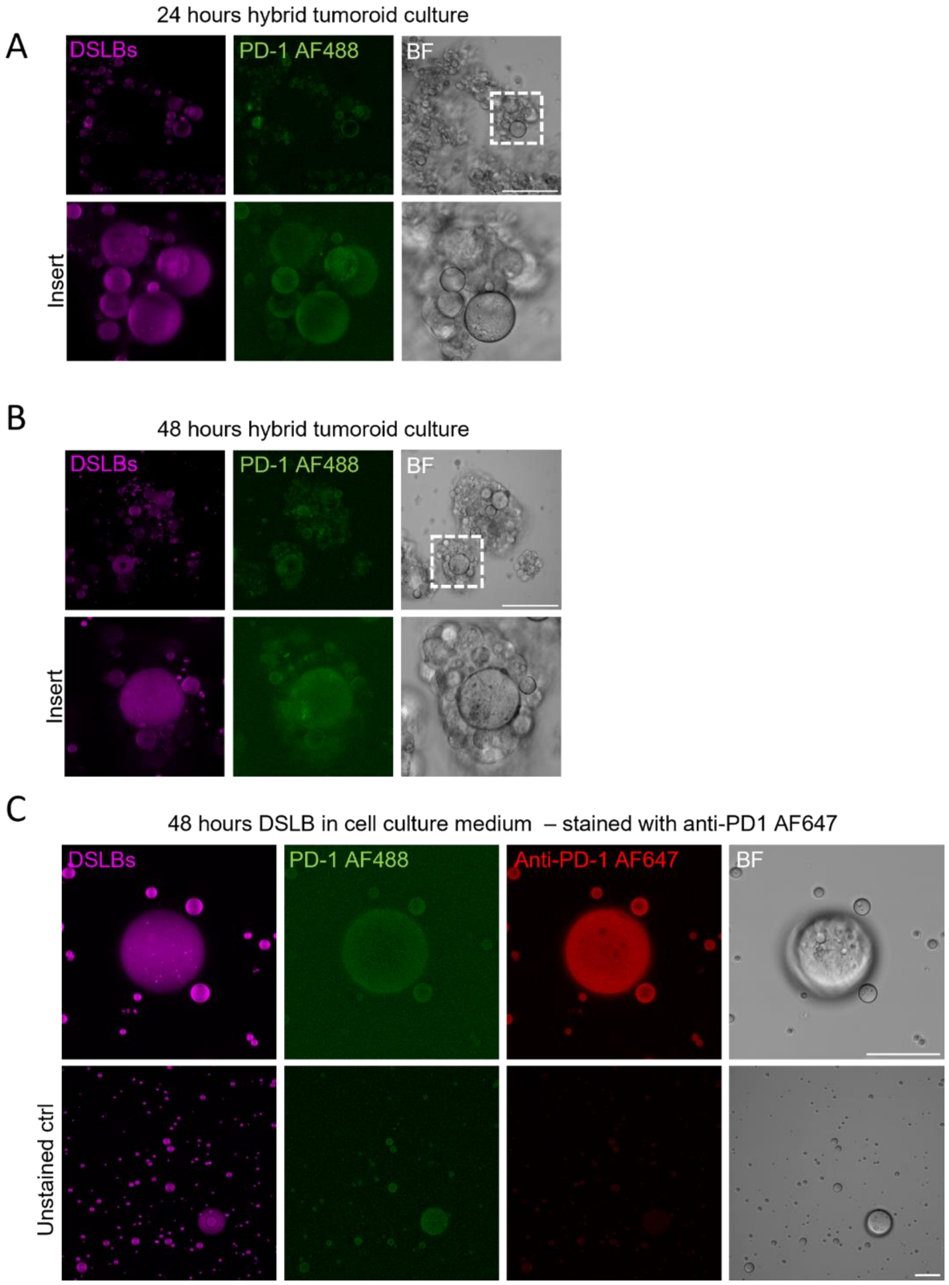

Figure S5: Stability of DSLB-protein functionalization. **A**) & **B**) Representative confocal microscopy maximal z-projections of tumoroids after 24 hours or 48 hours of culture. DSLBs were decorated with Alexa Fluor 488-labelled PD-1 and used in the formation of hybrid tumoroids. Scale bars are 150 µm. **C**) Enlarged confocal microscopy maximal z-projection of a DSLB decorated with fluorescent PD-1 after 48 hours of incubation in cell culture medium at 37°C. The stability of the protein was tested via staining with a specific anti-PD-1 antibody. Scale bars are 50 µm.

***Point 4:** With respect to the biological assays, the authors should include the controls in Figure 3 where both PD1 and CD2 are added in solution (without DSLBs) to clearly demonstrate a scaffolding effect. Also DCD40L should be added in solution as a control in Fig 2.*

We agree with the reviewer that addition of soluble ligands to the cultures are relevant controls. For this, we determined the approximate total concentration of ligands within the culture presented on the synthetic cell surface (verified by ELISA) and added an equivalent amount of soluble protein. For both systems, CD40L (as measured by Bfl-1 expression, see also reviewer 2, point 3) and PD1/CD2-presenting synthetic cells (as measured by CCL2 secretion), no comparable effects could be detected. This is mostly likely because presenting such receptor ligands on a membrane supports the formation of receptor clusters and drives avidity effects, both required for the formation of signaling competent interfaces, such as immune synapses, between immune and cancer cells in vivo.

We have added the respective controls in Figures S3F and S7 of the revised version of our manuscript and write on page 10, line 21 to page 11, line 2: "*Immuno-staining showed localization of CD40-enriched granules, similar to those observed between T cells and dendritic cells*[25]*, at the tumor-synthetic cell interface (**Fig. 2H**) and quantitative PCR together with western blot analysis revealed increased expression of the anti-apoptotic proteins Bfl-1, a known CD40 down-stream target gene expressed in Panc-1 cells*[26,27] *(**Fig. S3E,F**). Addition of soluble CD40L to the culture medium did not result in a comparable increase in expression (**Fig. S3F**).*"

And on page 16, lines 8-9: "*Importantly, addition of the soluble CD2 and PD-1 did not induce similar effects (**Fig. S7**).*"

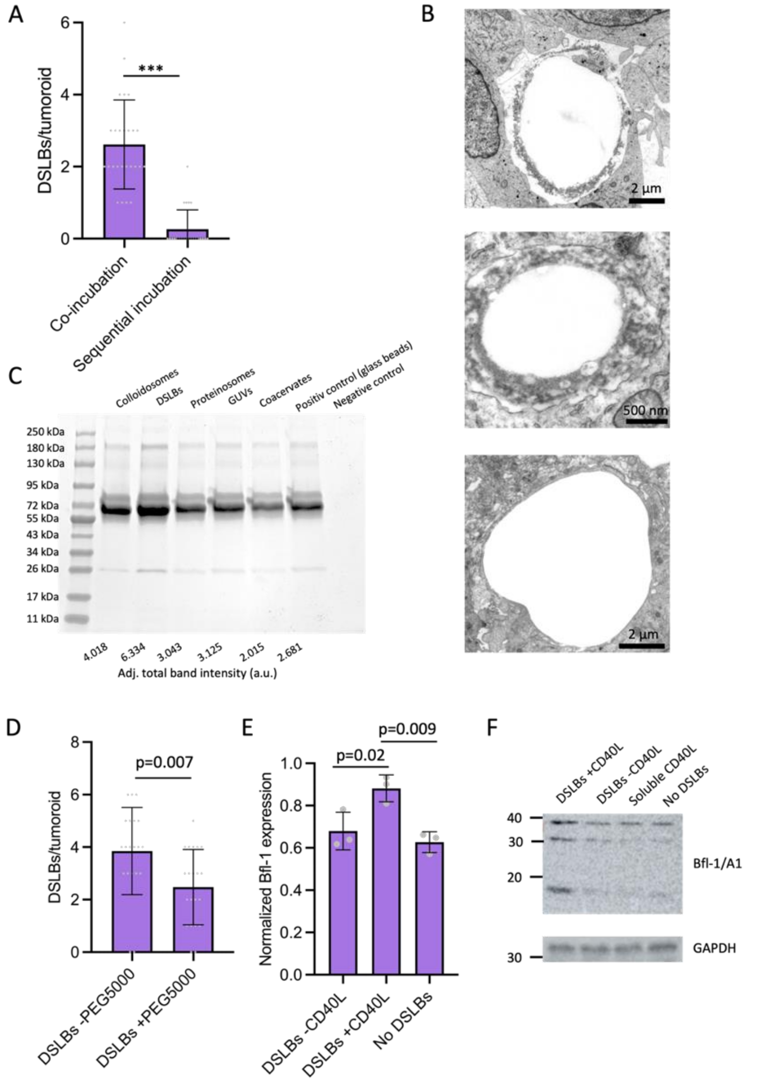

Figure S3: Self-assembly mechanisms of hybrid tumoroids. **A)** Quantification of DSLB-based synthetic cells integration into tumoroids formed from Panc-1 cells. DSLBs were either incubated with singularized synthetic cells for 48 hours (co-incubation), or added to pre-formed spheroids (sequential incubation). Results shown as mean ±SD from n>23, *** p<0.001 unpaired two-tailed t-test. **B)** Additional representative examples of the synthetic cell-cancer cell interface from TEM images, showing either the close contact between the synthetic cell membrane and the cell membrane or denser layers of surrounding ECM. **C)** SDS PAGE of serum opsonization levels from the synthetic cell panel incubated in 10% fetal bovine serum for 1 hour. Total band intensity for each lane is indicated in the bottom. **D)** Quantification of DSLB-based synthetic cells integration into tumoroids formed from Panc-1 cells either with plain DSLBs or DSLB produced with 10 mol% PEG5000 on the lipid bilayer surface. Results shown as mean ±SD from n>20, p values as indicated from unpaired two-tailed t-test. **E)** Quantitative PCR of Bfl-1 RNA levels in Panc-1 hybrid tumoroids formed with DSLBs presenting CD40L, plain DSLBs or no DSLBs. Results are shown from 3 separate replicates shown as mean ±SD, normalized to RNA levels of glyceraldeyde-3-phosphate dehydrogenase. P values as indicated from one-way ANOVA. **F)** Western blot analysis of Bfl-1 expression in Panc-1 hybrid tumoroids after culture with DSLBs presenting CD40L, with plain DSLBs, with plain DSLBs and 1 µg/mL soluble CD40L and DSLB free cultures. GAPDH was used as loading control.

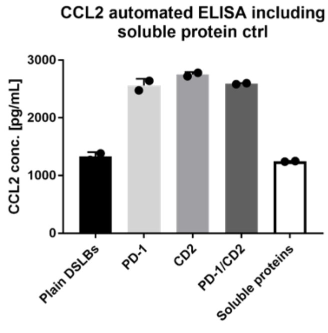

Figure S7: Enzyme-linked immunosorbent assay-based quantification of soluble CCL2 secreted from Panc-1 tumoroids with ART-TIMEs of varying composition after 48 hours of incubation. A soluble protein control was generated to rule out the impact of unbound protein left over from DSLB-functionalization. The concentration of soluble PD1 and CD2 was adjusted to 1.5 nM, reflecting the concentration range generated by ART-TIMES in Panc-1 tumoroids. Results shown as mean ±SD from 2 biological replicates measured in technical triplicates.

Point 5: *Furthermore, since only a fraction of the cancer cells is in contact with the artificial ones, I would expect a heterogeneous response of the tumoroid. The authors should try to analyze if there are two populations present.*

We thank the reviewer for pointing out this important aspect of heterogeneity in cellular responses within our hybrid tumoroids. To address this -and building upon our observations of trogocytosis-like events (see also response to reviewer 1, point 1)- we used membrane uptake as a proxy for contact formation between cancer cells and DSLBs. Specifically, we assessed the proportion of cancer cells that had been in contact with synthetic cells over the culture period by quantifying the uptake of fluorescent membrane parts after 48 hours. To this end, tumoroids were dissociated using trypsin and analyzed via flow cytometry. As controls, we included cancer cells cultured in tumoroids without synthetic cells (negative control) and cultures with liposomes of same membrane composition, known to undergo efficient uptake (positive control). Our data revealed that in hybrid tumoroids formed at 10:1 synthetic-to-natural cell ratios—the same ratio used across all our experimental conditions—the vast majority of cancer cells displayed a fluorescent membrane signal, indicating prior contact with a synthetic cell. Importantly, we also tested a series of initial seeding ratios (1:3, 1:5, 1:10) and observed that under these conditions every cell showed evidence of contact. In

contrast, at lower synthetic-to-natural cell ratios (1:10 or 1:1), not all cells made contact, as expected. This is consistent with our live imaging data (see Movie S2), which confirms that hybrid tumoroid assembly is highly dynamic and involves continuous remodeling, allowing broad distribution of contacts over time. Together, these data indicate that under our experimental conditions, nearly all cells experience contact with synthetic cells during the 48-hour culture period, reducing the likelihood of distinct subpopulations based on DSLB interaction. In the revised version of our manuscript, we now write on page 10, lines 3-10: *"Importantly, we also found evidence of trogocytosis-like behavior in which the cancer cells internalized small parts of the DSLB fluorescent membrane (**Fig. S4A**). By disintegrating the hybrid tumoroids after culture and quantifying the fluorescent membrane content in the cancer cells via flow cytometry, we observed that at a ratio of 1:10 cancer cells to synthetic cells, every cancer cell integrated some level of membrane fluorescence during the 48 hour culture period (**Fig. S4B**). The corresponding broad distribution of contacts indicates that under these conditions hybrid tumoroid assembly is highly dynamic and involves continuous remodeling. In contrast, not every cancer cell showed membrane fluorescence at ratios below 1:3."*

These results are now included in Figure S4 of the revised manuscript.

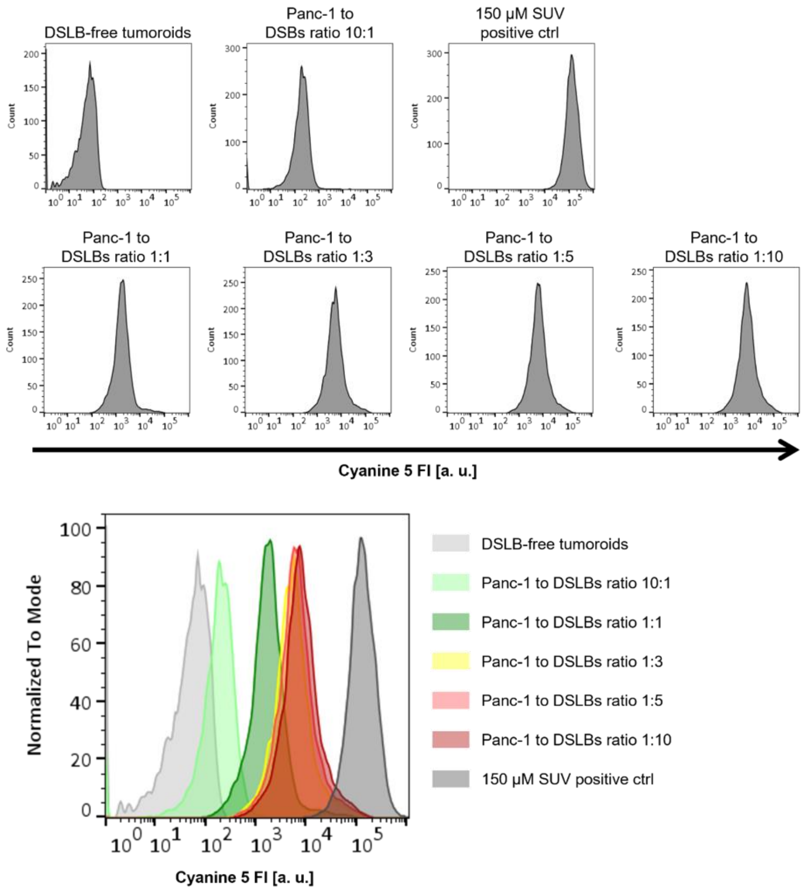

Figure S4: Contact events and membrane exchange between the Panc-1 cells and synthetic cells. Quantification of DSLB-membrane fluorescence intensity in Panc-1 cells of various hybrid tumoroid cultures. DSLBs featuring a membrane with a strong Cyanine 5 fluorescent signal were generated and used in the formation of hybrid tumoroids. Each condition was generated using 50 000 Panc-1 cells and varying numbers of DSLBs indicated by the Panc-1 to DSLBs ratio. After 48 hours of culture, the hybrid tumoroids were disassembled via trypsinization and subsequently fixed and stained for nuclei using Hoechst. Cyanine 5 fluorescent intensity in individual Panc-1 cells was analyzed via flow cytometry. DSLB-free tumoroids served as negative control and DSLB-free tumoroids cultivated for 48 hours in medium supplemented with 150 µM of the SUV stock solution utilized in DSLB generation were utilized as positive control.

Point 6: *The biological outcome is not very consistent. There is no real pattern to be observed from the effect of PD1, CD2 or the combination in Fig 3E. How can these results be explained? Based on the large variability, I am not sure if any biological conclusion can be drawn.*

We thank the review for pointing out that our description of the observed patterns was not evident enough and readers could appreciate a more detailed description of emergent effects in our analysis in Figure 3E and the separate quantification in Figure 3F. We observed several additive, synergistic or paradox signaling effects between PD1, CD2 and their combination, specifically for the phosphorylation status of ERK1/2 (T202/Y204, T185/Y187), p53 (S392), HSP60 and PRAS40 (T246). To point out one specific co-signaling axis, previous studies has demonstrated an interdependence between the phosphorylation status of p53 and HSP60 (https://doi.org:10.1038/cdd.2017.143 and https://doi.org:10.1074/jbc.M705904200). Our results now reveal that this concurrent and antagonistic phosphorylation, which is relevant for immune evasion, is initiated only when both T cell-related ligands PD-1/CD2 are presented in combination. An insight that could not be made before and enabled by our system.
We have now added additional description of these aspects and insights on page 15, lines 15-22: "*Importantly, differential effects with additive (e.g. ERK1/2 T202/Y204, T185/Y187), synergistic (e.g. p53 S392 and HSP60) or paradox (e.g. PRAS40 T246) phosphorylation were observed between PD1, CD2 and PD1/CD2 ART-TIMEs (**Fig. 3F**), highlighting the complex intracellular interplay induced by co-reverse signaling. Of note, some of these emergent effects, e.g. the inverse interplay between p53 and HSP60 phosphorylation, have been characterized before[36,37], suggesting that one co-signaling axis between PD1 and CD2 involves down regulation of HSP60, triggering increased mitochondrial import of pS392 phosphorylated p53.*"

Point 7: *Fig 1 C Could the authors indicate how many living cells are in the tumoroid; what is the ratio between artificial and living cells after the tumoroid has been fully formed?*

We thank the reviewer for pointing out that important information is missing in our manuscript. We initiate cultures with a 10:1 synthetic cell to natural cell ratio but not all synthetic cells are integrated into the final hybrid tumoroids, so this ratio is different after the hybrids have been formed. On average, one hybrid tumoroid contains 28 ±17 cancer cells, resulting in synthetic cell to natural cell ratios between 1:22 to 1:8 (for Figure 1C). Based on our light sheet imaging data, we now provide additional analysis on this aspect and corelate the number of synthetic cells in the tumoroids to the number of immune cells found in the tumors of PDAC patients. We found that from a volumetric comparison, hybrid tumoroids can cover the full spectrum of "cold" and "hot" PDAC tumors. As already mentioned for reviewer 1, point 1, hybrid tumoroids are preferentially formed from larger synthetic cells, potentially based on the more stable adhesion interface that can be formed and that is required for hybrid formation.
We now have updated this relevant information in the revised version of our manuscript on pages 5, line 24 to page 6, line 3: *"Quantification of the number of integrated DSLB- and colloidosome-based synthetic cells per tumoroid for all cell lines revealed the highest integration rates in Panc-1 and Capan-1, followed by BT-747 tumoroids (**Fig. 1C**). Based on this approach, synthetic cell to natural cell ratios of 1:22 to 1:8 could be achieved."*

We further added Figure S6 to the revised version of our manuscript, showing the results of our volumetric analysis and now write on page 14, lines 13-18: *"We performed volumetric 3D light sheet microscopy of hybrid tumoroids and compared the volume of the synthetic cell population within them to reported values from histological analysis from PDAC patients (**Fig. S6**). We found that synthetic cells in hybrid tumoroids can represent similar volume fractions as infiltrating immune cells in PDAC tumors. This further highlights the suitability of our hybrid tumoroids to mimic immune cell infiltration."*

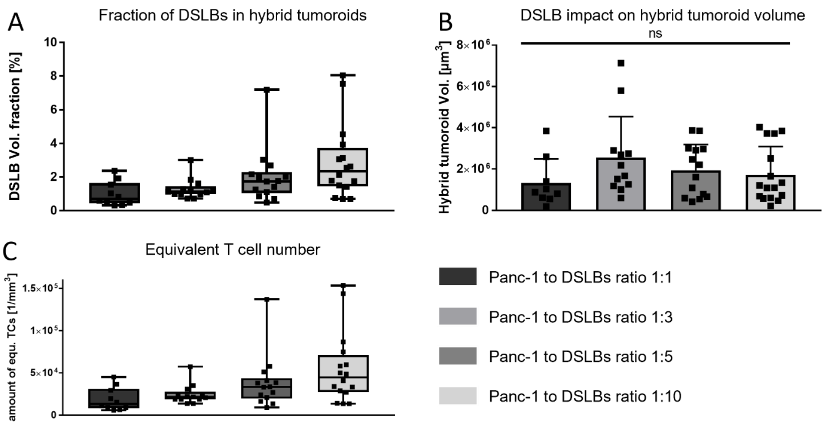

Figure S6: Impact of DSLB quantity used in hybrid tumoroid formation. **A)** Quantification of the volume fraction of a tumoroid consisting of integrated DSLB-based synthetic cells. Tumoroids were formed from 50 000 Panc-1 cells and varying numbers of DSLBs indicated by the Panc-1 to DSLBs ratio. Results are shown as a box plot comprised of min and max whiskers, all data points, upper and lower quartiles and median from n>10. **B)** Quantification of the total volume of hybrid tumoroids composed of Panc-1 cells and integrated DSLB-based synthetic cells. Results shown as mean +SD from n>10, ns p>0.05 one-way ANOVA. **C)** Quantification of the number of T cells equivalent to the density of integrated DSLB-based synthetic cells in various hybrid tumoroids. The total DSLB volume per tumoroid was used to calculate the number of T cells and normalized to a theoretical tissue volume of 1 mm^3. A T cell was estimated as a spherical object with a diameter of 10 µm. Results are shown as a box plot comprised of min and max whiskers, all data points, upper and lower quartiles and median from n>10.

Point 8: *I can't find movie S2.*
We thank the reviewer for point out that a problem seems to have occurred during file upload. We have re-uploaded movie S2 into the submission system.

Point 9: *Line 15 page 10 fig 3F should be 2F*
We thank the reviewer for pointing out this typo which has been corrected in the revised version of our manuscript.

Reviewer #2:

In this manuscript, the authors present an innovative approach to generate 3D cancer cell cultures by systematically evaluating different synthetic cell mimetics as scaffolds/inducers of 3D culture formation. They assess the underlying principles and utilize this novel model system to study tumor receptor co-signaling. The study reveals new insights into tumor immunosuppression via PD1 and CD2, provides relevant data using a bi-specific T cell engager, and correlates the signature with overall survival in pancreatic carcinoma patients. This well-

written manuscript demonstrates significant innovation and potential for studying cancer-immune interactions in 3D cell cultures.

We thank the reviewer for his/her encouraging assessment of our manuscript and would like to address the suggestions for further improvement in the following point-by-point response.

Point 1. *While the authors establish the importance of adhesion molecules in 3D culture formation, their data relies solely on transcriptomic analyses. Protein expression data for E-cadherin, CD29, and key expressed integrins would provide valuable validation.*

We agree with the reviewer that there is typically a poor correlation between RNA levels and protein expression and our study would profit from additional validation on the protein level expression of the relevant adhesion proteins. In our revised manuscript version, we now include western blot analysis to determine differences in adhesion protein expression between the different cell lines. In this comparison, we find good correlation for almost all adhesion proteins on the mRNA and protein level. Most importantly, this analysis is in line with our argumentation that a balance between extra- and intercellular adhesive proteins directs the integration efficacy of synthetic cells into hybrid tumoroids. These results have been added as Figure S1B and described on page 5, lines 8-11 in our revised manuscript version: *"The cells were selected based on the expression (mRNA and protein level) of extra- and intercellular adhesion proteins (**Fig. S1A,B**), covering fast and slow growing cultures as well as representing high immunogenic (breast) and low immunogenic (PDAC) cancers."*

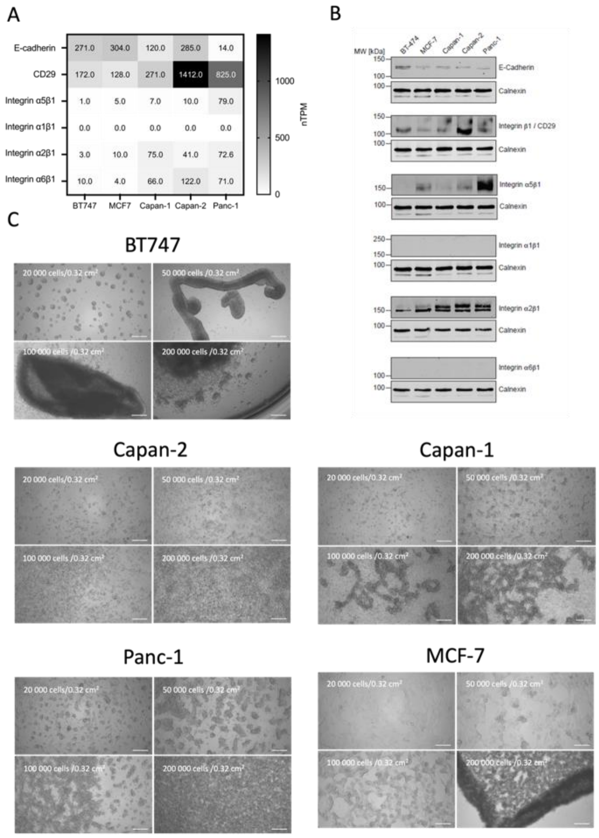

Figure S1: 3D cell culture cell lines. **A**) Normalized transcripts per million data for selected intercellular (cadherin) and extracellular (integrins) adhesion proteins in of the cell lines used in this study. Data was derived from the human protein atlas (proteinatlas.org). **B)** Western Blot analysis of cell line lysates using antibodies specific for the selection of inter- and extracellular adhesion proteins as indicated. Anti-Calnexin antibodies were used as loading control. Representative example from two biological replicates. **C)** Bright field microscopy data of the

five cell lines used in this study, plated at various densities on low adhesive surfaces to induce 3D culture formation. Scale bars are 100 µm.

Point 2. *The authors should address the long-term stability of their model system, including maximum culture duration and whether prolonged culture affects architecture and/or cellular composition.*

The reviewer raises a relevant point pertaining to the long-term culture of hybrid tumoroids, which we agree, has only been peripherally addressed in our initial manuscript version. For our revised manuscript version, we have now performed further analysis of the hybrid tumoroid architecture and cellular composition over prolonged culture periods. For this we applied light sheet volumetric imaging to determine the morphological maturation of the tumoroids, the integrity of the synthetic cells, specifically their membrane, and their cellular composition. We found that while the tumoroids undergo further morphological maturation in a 7-day culture period, forming a central lumen and undergoing further growth, the synthetic cells remain stably integrated within the cellular architecture. Moreover, the DSLB membrane remains intact and continuous during the full period. This underscore the stability of the formed hybrids and, combined with the stable coupling of the receptor ligands to their surface (see reviewer 1, point 3), demonstrates that our system is compatible with experiments setups requiring long term culture.
These results have been added as Figure S2C and described on page 6, lines 6-19 in our revised manuscript version: *"Importantly, we observed retention of colloidosomes and DSLBs within the tumoroid for a 7-day growth period, indicating stable incorporation and retention within the 3D architecture (**Fig. S2C**). We found that the DSLB membrane architecture remains overall intact during this prolonged culture period and while the tumoroids undergo further morphological maturation (e.g. formation of a central lumen), the synthetic cells remain integrated within the cellular architecture."*

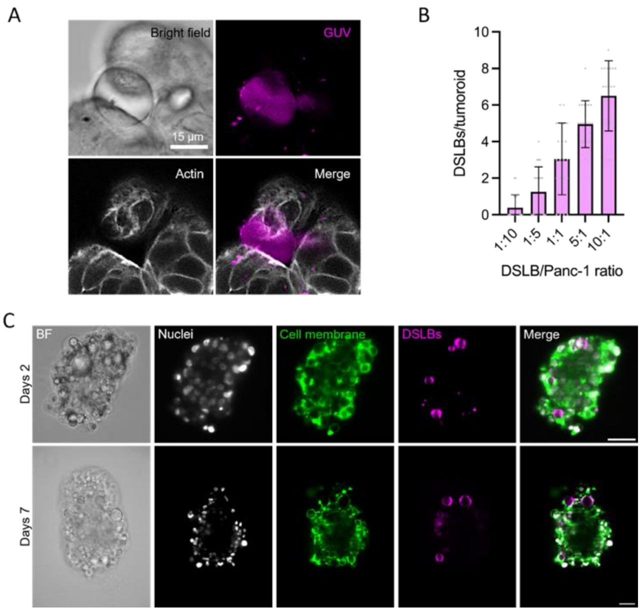

Figure S2: Stable integration of synthetic cells into tumoroids. **A)** Representative confocal microscopy maximal z-projections of Panc-1 tumoroid integrating a GUV as a synthetic cell model after 48 hours of culture. **B)** Quantification of DSLB-based synthetic cells integration into 3D cultures formed from Panc-1 cells after 48 hours of co-culture in various synthetic cell to Panc-1 ratios. Results shown as mean ±SD from n>21. **C)** Representative light sheet microscopy images of DSLB-based synthetic cells integrated into Panc-1 tumoroids 2 days and 7 days

after formation. Cells were stained for membrane (wheat germ agglutinin, green) and nuclei (Hoechst, grey). Scale bar is 50 µm.

Point 3. *The CD40 experiment demonstrates Bfl1 transcription but not protein expression. Given that transcription and translation don't always correlate directly, analyzing Bfl1 protein expression would strengthen these findings.*

Similar to the point raised by reviewer 1, point 1, and similar to reviewer 1 point 4, we agree that providing further proof on the protein level for Bfl1 could improve our study. Based on the reviewer's suggestion, we performed further western blot validation on Bfl1 expression, which verified our previous qPCR analysis. Presentation of CD40L on DSLBs specifically increased Bfl1 expression levels, further demonstrating that synthetic cells and living cells can form competent signaling interfaces. Importantly, control experiments with soluble CD40L (also see reviewer 1, point 4) showed that the presentation of the CD40L molecule on the DSLB surface is required to initiate signaling, highlighting the role of the membrane in our synthetic cell system. These results have been added as Figure S3F and described on page 11, lines 1-2 in our revised manuscript version.

*"Addition of soluble CD40L to the culture medium did not result in a comparable increase in expression (**Fig. S3F**)."*

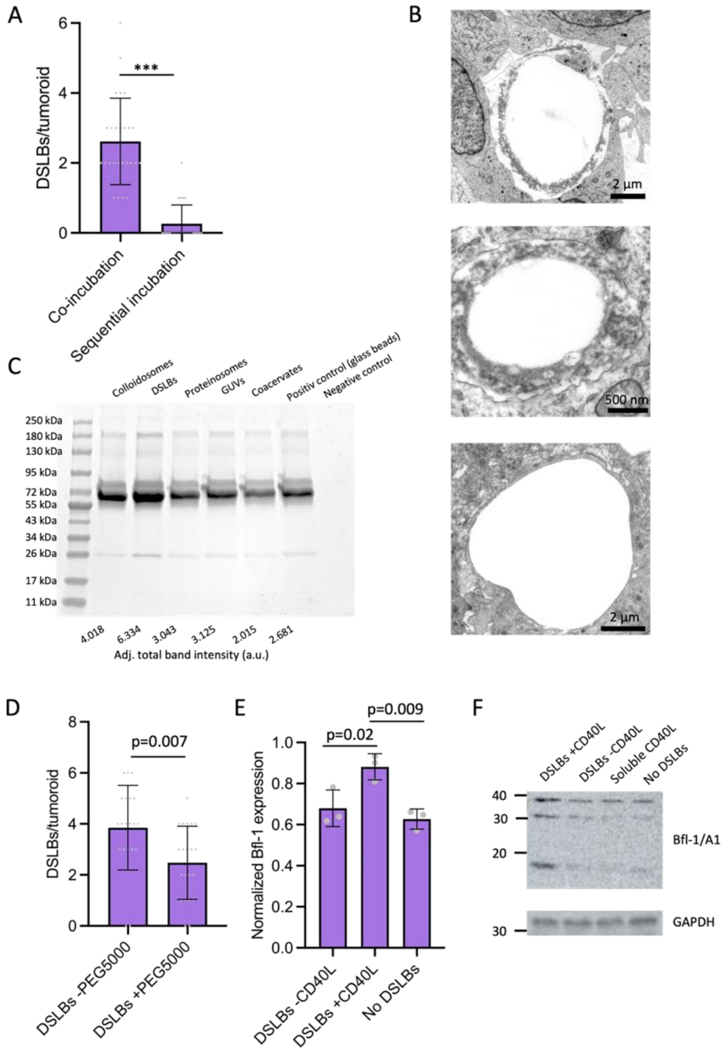

Figure S3: Self-assembly mechanisms of hybrid tumoroids. **A)** Quantification of DSLB-based synthetic cells integration into tumoroids formed from Panc-1 cells. DSLBs were either incubated with singularized synthetic cells for 48 hours (co-incubation), or added to pre-formed spheroids (sequential incubation). Results shown as mean ±SD from n>23, *** p<0.001 unpaired two-tailed t-test. **B)** Additional representative examples of the synthetic cell-cancer cell interface from TEM images, showing either the close contact between the synthetic cell membrane and the cell membrane or denser layers of surrounding ECM. **C)** SDS PAGE of serum opsonization levels from the synthetic cell panel incubated in 10% fetal bovine serum for 1 hour. Total band intensity for each lane is indicated in the bottom. **D)** Quantification of DSLB-based synthetic cells integration into tumoroids formed from Panc-1 cells either with plain DSLBs or DSLB produced with 10 mol% PEG5000 on the lipid bilayer surface. Results shown as mean ±SD from n>20, p values as indicated from unpaired two-tailed t-test. **E)** Quantitative PCR of Bfl-1 RNA levels in Panc-1 hybrid tumoroids formed with DSLBs presenting CD40L, plain DSLBs or no DSLBs. Results are shown from 3 separate replicates shown as mean ±SD, normalized to RNA levels of glyceraldeyde-3-phosphate dehydrogenase. P values as indicated from one-way ANOVA. **F)** Western blot analysis of Bfl-1 expression in Panc-1 hybrid tumoroids after culture with DSLBs presenting CD40L, with plain DSLBs, with plain DSLBs and 1 µg/mL soluble CD40L and DSLB free cultures. GAPDH was used as loading control.

Point 4. *Additional replicates for the experiments shown in Figures 3J and 3K would enhance statistical significance.*

We agree with the reviewer that our killing assays with bispecific T cell engagers and primary T cells as well as the ELISA-based quantification of key effector cytokines from these cultures could profit from further replicates. The p value based on our experiments with several biological and technical replicates of p=0.0003, p=0.0001 and p<0.0001 now indicate high statistically significant difference between control hybrid tumoroids and those with PD-1/CD2 ART-TIMEs. Based on the reviewer's suggestion (also in reviewer 2, point 5) we have now performed several experiments, also with other T cell to cancer cell ratios, and provided more statistical analysis between the individual conditions. This additional analysis further supports our observations that CD2 can co-signal with PD-1 to drive cancer immune regulation. Our new results and analysis have been added as Figure 3K in our revised manuscript version.

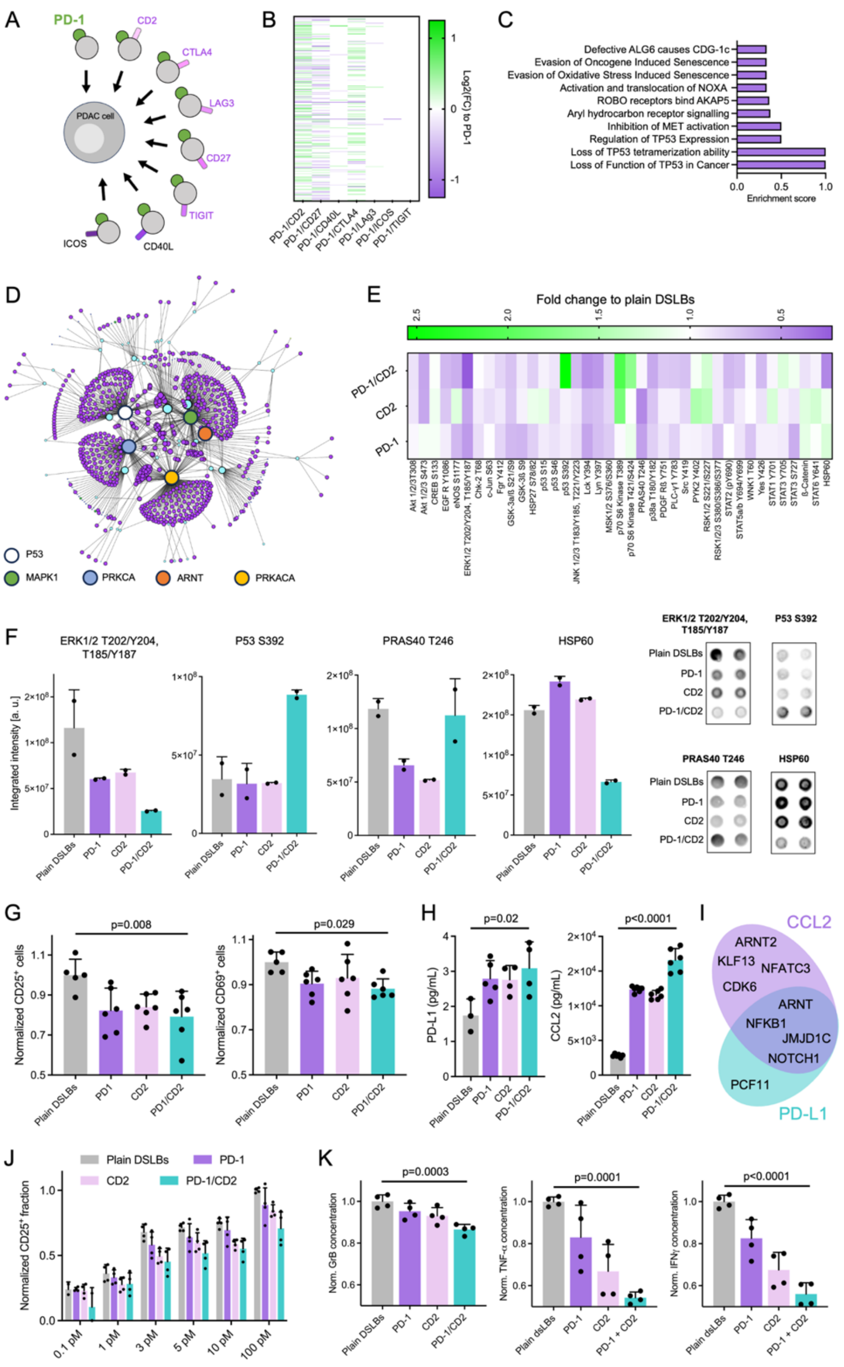

Figure 3 Artificial tumor immune microenvironments. **A)** Schematic representation of the ART-TIME screening approach and the panel of immune ligands tested. **B)** Differential gene expression analysis of Panc-1 tumoroids with integrated ART-TIMEs presenting varying combinations of immune ligands. DEGs compared to ART-TIMEs with PD-1 only are shown to derives transcriptional changes based on receptor co-signaling. **C)** Pathway enrichment analysis from PD-1/CD2 DEGs in C. Pathways with the ten highest enrichments scores are shown. **D)** Gene network analysis from PD-1/CD2 DEGs. 5 nodes with the highest degrees are highlighted. **E)** Heat map of protein phosphorylation analysis from Panc-1 tumoroids with ART-TIMEs of PD-1, CD2 and PD-1/CD2. Phosphorylation levels are shown as compared to hybrid tumoroids with plain DSLBs. **F)** Four exemplary protein phosphorylation levels from E, showing synergistic, paradox or *de novo* effect with PD-1/CD2 co-presentation. Results shown as mean from two antibody spots pooled from forty technical replicates. **G)** Flow cytometry quantification of the fraction of CD25 and CD69 expressing primary human CD8 T cells incubated with pre-conditioned medium of Panc-1 tumoroids with varying ART-TIME compositions. Results are shown from n=3 donors as mean ⍰SD from two replicates each. **H)** Enzyme-linked immunosorbent assay-based quantification of soluble PD-L1 and CCL2 secreted from Panc-1 tumoroids with ART-TIMEs of varying composition after 48 hours

of incubation. Results shown as mean SD from 2 biological replicates measured in technical triplicates. **I)** Ven diagram of PD-L1 and CCL2 transcription factors identified as DEGs in PD-1/CD2 ART-TIME tumoroids. **J)** Flow cytometry quantification of the fraction of CD25 expressing primary human CD8$^+$ T cells incubated with ART-TIME containing Panc-1 3D cultures for 12 hours in the presence of increasing bispecific T cell engager concentrations. Results are shown from n=2 donors as mean SD from two replicates each. **K)** Enzyme-linked immunosorbent assay-based quantification of three effector cytokines secreted from primary human CD8$^+$ T cells incubated with Panc-1 tumoroids with ART-TIMEs of varying composition after 12 hours of incubation with 5 pM bispecific T cell engager concentration. Results shown as mean SD from 2 biological replicates measured in duplicates. p values are indicated from one-way ANOVA statistical testing.

Point 5. *While the authors note differences in T cell activation between groups (plain DSLC, CD2, PD-1, CD2/PD-1) but no statistical difference in killing potency, their analysis uses only one T cell:target ratio (5:1). Testing multiple ratios, particularly sub-optimal ones, would provide more comprehensive insights.*

The reviewer brings up a-very good suggestion to further reveal potential differences in killing efficacy between our control and ART-TIME containing hybrid tumoroids by testing also sub-optimal T cell to target cell ratios. Following this suggestion, we now performed additional bispecific T cell engagers killing assays with plain DSLB, PD1, CD2 and PD1/CD2 hybrid tumoroids but with T cell to target cell ratios of 1:2 and 2:1, which are both far away from the optimal performance of this assay. We resolved killing efficacy again by LDH release assays but as in our experiments with the 5:1 condition, we could not resolve any significant difference between the ART-TIME configurations. In fact, both additional ratios did not deliver any good killing efficacy. These new results, together with those presented in the initial version of our manuscript, point to the fact that the PD1/CD2 co-signaling-based phenotypic adaptation does significantly impact on the cancer cells´ ability to inhibit T cell function but this does not fully translate into a difference in killing efficacy, at least in this *in vitro* set up. However, this might not be too surprising since these are only two proteins presented on T cells and will therefore likely not recapitulate the full effect size. However, the significant differences in cytokine release do confirm our principal concept that synthetic cells can be stably integrated into tumoroids and be applied to mimic cancer-T cell interactions in a very systematic and signaling-competent manner. In line with the reviewer's view, we do believe that these new results should be include in the revised version of our manuscript to further support argumentation basis on the killing efficacy.

The new data have been described on page 17, lines 1-4 in our revised manuscript version: *"We observed dose-dependent killing in a 0.1-100 pM range of bispecific engagers but did not resolve any significant differences in their efficacy when comparing hybrid tumoroids containing plain DSLBs and PD-1/CD2 ART-TIMEs, even at varying T cell to cancer cell ratios (**Fig. S8A,B**)."*
And added as Figure S8A,B:

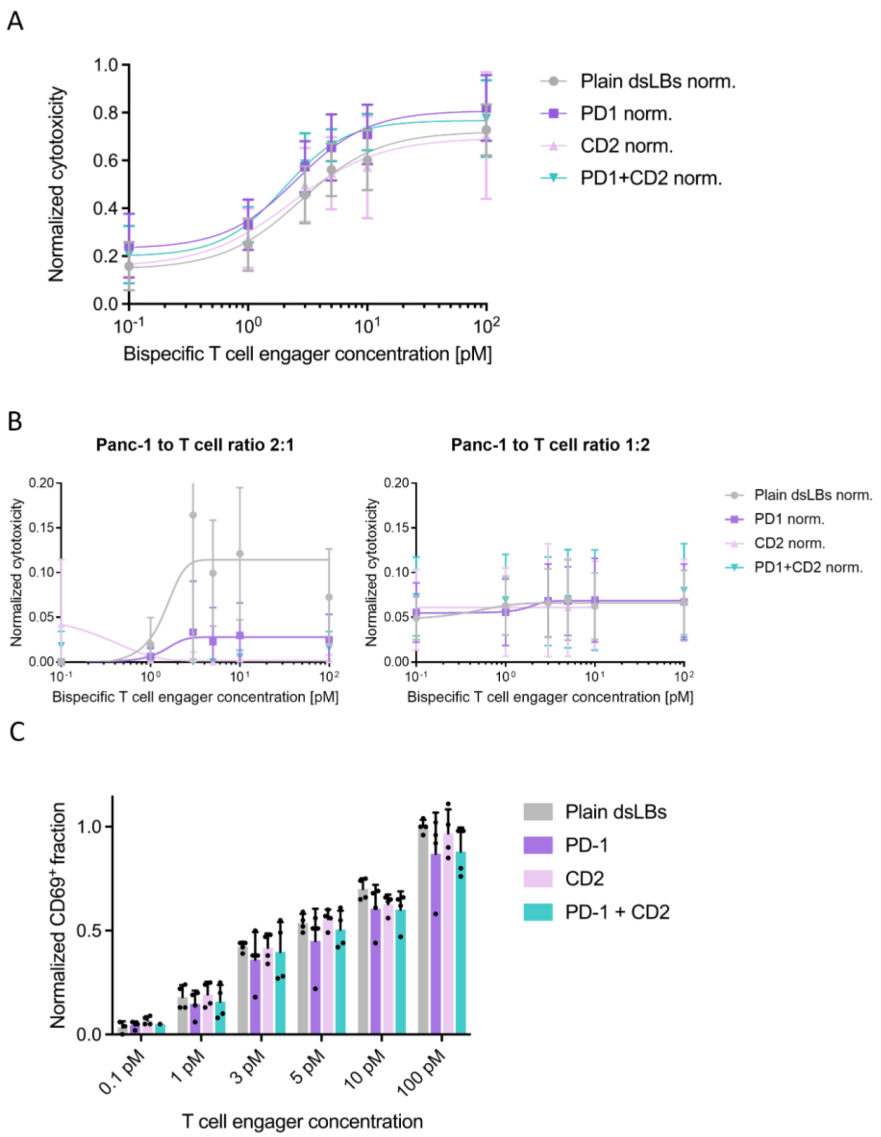

Figure S8 Bispecific T cell engager-mediated killing of hybrid tumoroids. **A)** Lactate dehydrogenase release assay for quantification of killing of ART-TIME containing Panc-1 hybrid spheroids by primary human CD8 T cells in a bispecific T cell engager dilution series. T cells were incubated with hybrid tumoroids preformed for 48 hours at a 5:1 T cell to cancer cell ratio. Results are shown from n=2 donors as mean ±SD from two replicates each. **B)** Lactate dehydrogenase release assay for quantification of killing of ART-TIME containing Panc-1 hybrid spheroids by primary human CD8 T cells in a bispecific T cell engager dilution series under suboptimal conditions. T cells were incubated with hybrid tumoroids preformed for 48 hours at a 2:1 or 1:2 T cell to cancer cell ratio. Results are shown from n=2 donors as mean ±SD from two replicates each. **C)** Flow cytometry quantification of the fraction of CD69 expressing primary human CD8 T cells incubated with ART-TIME containing Panc-1 3D cultures for 12 hours in the presence of increasing bispecific T cell engager concentrations. Results are shown from n=2 donors as mean ±SD from two replicates each.

Point by point response:

Reviewer #1 (Remarks to the Author):

The authors have provided novel data and clear answers to the questions raised. Based on this additional information I feel the paper is now suited for publication

Reviewer #2 (Remarks to the Author):

The authors have adequately addressed all concerns raised in the previous review. I have no additional comments or issues to be addressed.

We thank the reviewers for their time and effort to reviewer our manuscript and significantly improve it!
